# Effects of Long-Term Heavy Metal Exposure on Oral Microbial Antibiotic Resistance Genes of Residents in the Mining and Smelting Area

**DOI:** 10.3390/microorganisms13122814

**Published:** 2025-12-10

**Authors:** Huan Li, Keke Yang, Hongling Liu, Susu Cao, Yuanyuan Bao, Lu Feng, Li Zhang, Jingping Niu, Tian Tian

**Affiliations:** 1 School of Public Health, Lanzhou University, Lanzhou 730000, China; lhuan2023@lzu.edu.cn (H.L.);; 2 School of Stomatology, Lanzhou University, Lanzhou 730000, China

**Keywords:** heavy metal, resistome, oral bacteria, network analysis, metagenomic sequencing

## Abstract

Growing evidence highlights the role of heavy metals in driving the co-selection of an-tibiotic resistance genes (ARGs), and the human oral cavity is an important reservoir of ARGs. This cross-sectional study investigated the effects of heavy metal exposure on human oral microbiota and ARGs, collecting buccal mucosal and blood samples from residents in a heavy metal-contaminated area (Baiyin City) and a non-contaminated area (Yuzhong County, Lanzhou City). The results showed heavy metal exposure is associated with altered alpha and beta diversity of bacteria and ARGs in human oral cavities, with bacterial compositional shifts being the main factor in ARG variation. Metagenomic analysis revealed heavy metal exposure is linked to modifying the interactions in the bacterial community and between ARGs and metal resistance genes (MRGs), shown by simplified topological structures in bacterial and resistome networks, along with enhanced positive correlations among nodes. *Neisseria*, *Haemophilus*, *Morococcus*, *Streptococcus*, *Staphylococcus*, and *Mycobacteroides* as potential hosts for resistance genes in human oral cavity. Furthermore, blood metal quantification revealed distinct associations with resistance patterns. This study demonstrates significant associations between environmental heavy metal exposure and the oral resistome and emphasizes the role of bacterial community composition.

## 1. Introduction

The rate of global antibiotic consumption has soared by 46% since 2000 to 2018 [1], with projections indicating antimicrobial resistance could take 10 million lives by mid-century [2], and this trend makes antibiotic resistance a defining challenge to global health security. Antibiotic resistance is primarily mediated by antibiotic resistance genes (ARGs), which confer resistance to antimicrobial agents in microorganisms through diverse biochemical mechanisms [3,4]. ARG emergence follows two principal pathways: spontaneous mutations and horizontal gene transfer (HGT). Mutations have generally been found in genes not classed as ARGs and cannot transfer among bacteria. In contrast, HGT-mediated ARG dissemination by mobile genetic elements (MGEs) [5,6] can enable the potential dissemination of ARGs from symbiotic to pathogens, which could facilitate the spread of antibiotic resistance to clinically relevant strains [7,8]. Since ARGs are a worldwide threat, it is crucial to identify them in specific microbiomes. ARGs have been detected in both neonatal and adult oral cavities [9,10], and conferring resistance to tetracycline and amoxicillin are prevalent in oral samples [4,11]. The oral microbiome has been recognized as a significant ARG reservoir [3]. As an important component for human health [12], complex interactions of oral microbial communities help the host counteract adverse stimuli, and microbial metabolites from this ecosystem can enter the bloodstream [13]. Thus, dysbiosis within this ecosystem could be a cause of oral and generalized disorders [14,15,16]. As pathogenic bacteria can acquire resistance via HGT, oral bacterial resistance may trigger systemic antibiotic-resistant infections [17]. For example, resistant oral *streptococci* (e.g., to β-lactams, clindamycin, erythromycin) are implicated in infective endocarditis [3,18].

Oral microbiome dynamics are modulated by complex signals, including host and external environmental factors [19]. For instance, environmental heavy metals can be introduced into the human oral cavity via the food chain and disrupt the oral microenvironment and function, posing multiple health risks [20,21]. Emerging evidence suggests that there was a significant change in human oral microbial composition following prolonged exposure to heavy metals, characterized by reduced *Streptococcus* and elevated *Fusobacterium*, which correlated with an increased rate of oral diseases like lichen planus [22]. The assignment to exposure and control groups was confirmed by contemporaneous soil heavy metal data. Furthermore, many studies have found that metals are linked to ARGs through co-selection mechanisms. Metals promote the emergence of antibiotic resistance via co-resistance, cross-resistance, and co-regulation [23,24]. For example, co-selection of ARGs has been observed in soil fauna gut microbiomes under heavy metal exposure [25]. There is also a report that metals from bamboo exert a substantial influence on pandas’ gastrointestinal bacterial communities and resistance genes [26]. Besides that, co-occurring signals between ARGs and metal resistance genes (MRGs) are common in human pathogens [27].

As a repository of ARGs and maintaining direct contact with the external environment, the human oral microorganisms are susceptible to external influences, which highlights the comprehensive mapping of ARGs and MRGs within the human oral microbiome [28]. Existing research regarding the effect of heavy metals on oral microbial ARGs mainly focused on dental materials used in medicine [29]. Primate experiments have shown that mercury (Hg) exposure in dental amalgams facilitates the acquisition of antibiotic resistance in the oral microbiome [30]. However, how environmental heavy metal exposures (e.g., from mining, pollution) impact human oral microbial ARGs remains unclear. As mining activities release metals into ecosystems [31] and accumulate in residents via food chains, it is critical to research this issue. A typical example is Baiyin City, a historic mining hub in China, which has suffered severe heavy metal pollution from mining operations over the past century [32], and metals from soil, water, and air are absorbed through dietary exposure, resulting in bioaccumulation in residents [33,34,35]. We therefore conducted a cross-sectional study involving residents from the contaminated area in Baiyin City as the exposure group, and residents who lived 70 km away in Yuzhong County, Lanzhou City, as the control group. The control site shares similar lifestyles, dietary habits, and economic conditions with the exposure group, but has negligible heavy metal contamination. By comparing human oral microorganisms and ARGs between these two groups, our study aimed to: (i) characterize the oral bacteria and ARGs profiles in residents under environmental heavy metal exposure and (ii) trace potential hosts of resistance genes within the human oral microbiome.

## 2. Materials and Methods

### 2.1. Study Population and Sampling

This metagenomic analysis utilized archived biological samples from a previously established and well-characterized core cohort of 137 participants. The recruitment of participants from Baiyin City (exposure area) and Yuzhong County (control area), the sample collection procedures (including the collection of peripheral blood, two independent aliquots of buccal mucosa samples, and questionnaires), and the inclusion criteria have been described in detail elsewhere [22]. In brief, all participants were permanent residents of their respective regions for over ten years, had refrained from antibiotic use for at least three months before sampling, and were free of oral diseases. The core cohort was defined by the availability of complete paired data (buccal mucosa samples and questionnaires). Two aliquots of buccal samples were initially collected, and the first set of samples was used in previous analyses to help define the cohort and for preliminary exploration (16S rRNA gene sequencing). A pre-existing blood metal analysis from a subset of this cohort (*n* = 79) had established a significant exposure contrast between the two regions, confirming their suitability as exposure and control groups. For this study, we utilized the second aliquot of buccal samples of the whole cohort (*n* = 137). Samples were excluded primarily due to insufficient DNA concentration for constructing high-quality metagenomic libraries. Consequently, only 37 samples (26 in exposure group, 11 in control group) qualified for the final analytical dataset. Since pre-existing blood metal data were only available for 15 of the 26 exposure individuals and 2 of the 11 control individuals within this subset, we performed a proxy analysis to verify whether the established exposure contrast was maintained. We compared the available data from the sequenced exposure individuals (*n* = 15) against all available data from the historical control group (*n* = 16, from the aforementioned sub-cohort of 79), and confirmed that the exposure group individuals maintained elevated blood metal levels (*p* < 0.05).

### 2.2. DNA Extraction and Metagenome Sequencing

The DNA was isolated from sterile cotton swabs using the FastPure Stool DNA Isolation Kit (Magnetic Bead) (MJYH, Shanghai, China), following the manufacturer’s guidelines. DNA purity was assessed using NanoDrop2000 (Thermo Fisher Scientific, Waltham, MA, USA) and integrity was confirmed on a 1% agarose gel. Subsequently, the DNA was sheared to 350 bp fragments using a Covaris M220 (Gene Company Limited, Hong Kong, China). The paired-end library was created with NEXTFLEX^®^ Rapid DNA-Seq (Bioo Scientific, Austin, TX, USA) and sequenced on an Illumina NovaSeq 6000 (Illumina Inc., San Diego, CA, USA) at Majorbio Bio-Pharm Technology Co., Ltd. (Shanghai, China). To ensure high-quality sequences, adapter sequences, and low-quality reads (Q-score < 20 or length < 50 bp) were trimmed using fastp (v0.20.0) [36]. Reads aligning with the human genome were filtered out using BWA (v0.7.17) [37]. The resulting high-quality microbial reads were compiled using Megahit (v1.1.2) [38], retaining at least 300 bp contigs as the final assembly output.

### 2.3. Gene Taxonomy and Functional Annotation

Gene prediction was performed using Prodigal v2.6.3 (https://github.com/hyattpd/Prodigal, accessed on 28 December 2023) [39], with Open Reading Frames (ORFs) at least 100 bp, followed by translation to amino acid sequences. These sequences were developed into a nonredundant gene catalog using CD-HIT (v4.7) (90% sequence identity, 90% coverage) [40]. Gene abundance was quantified by aligning high-quality reads to the catalog with SOAPaligner (v2.21) (95% identity threshold) [41]. Species identification and functional characterization were performed by aligning the non-redundant genes against reference databases using Diamond (v2.0.15) [42] with the following database-specific annotations: NR (taxonomic classification), CARD (ARGs) [43], BacMet (MRGs) [44], and MGE [45]. To ensure annotation reliability, stringent thresholds (*E*-value of <1 × 10^−5^, identity ≥ 70%, alignment length ≥ 50) were applied. Gene expression levels were quantified using transcripts per million (TPM), calculated as:

$$TPM=\frac{{(R}_{i}/L_{i})\times 10^{6}}{\sum_{1}^{n}{(R}_{i}/L_{i})}$$

where $R_{i}$ represents the number of reads mapped to the gene *i*; and $L_{i}$ denotes the length of gene *i* (bp).

### 2.4. Statistical Analyses

We calculated comprehensive analyses of microbial and resistome diversity using R (v4.4.2). Alpha diversity indices for bacterial communities, ARGs, and MRGs were compared between groups using Wilcoxon rank-sum tests, with statistical significance determined at a Benjamini-Hochberg corrected *p*-value threshold of 0.05. To examine compositional differences of bacteria and ARGs, we performed principal coordinate analysis (PCoA) based on Bray-Curtis or Jaccard distances and assessed their statistical significance through analysis of similarity (ANOSIM) and multivariate analysis of variance (Adonis), respectively. Multivariate models were adjusted for demographic (gender, age), socioeconomic (occupation, income), and behavioral (smoking, drinking, mobility status) covariates. Acheson distance after clr transformation using absolute counting is used as a supplementary analysis. We also used Procrustes analysis to infer correlations between resistance genes and bacterial communities and mobile genetic elements. Bacterial communities and resistance gene subtype co-occurrence networks were established through the integrated network analysis pipeline (iNAP) [46], implementing random matrix theory (RMT). Associations were assessed by Spearman’s rank correlation coefficients, only considering elements with occurrence frequency ≥ 50%. The resulting networks were visualized using Gephi (v0.10.1) or Cytoscape (v3.10.2) software. Contig-level co-localization of ARGs, MRGs, and MGEs was identified through Prokka (v1.14.6) and screening against specialized databases. Taxonomic assignment of contigs carrying these genes was based on alignment of inherent 16S rRNA sequences to the NCBI database. In addition, a Mantel test was performed to assess associations of blood metal levels with co-occurrence networks module eigenvalues (*n* = 15 samples), examining heavy metal effects on network architecture.

## 3. Results

### 3.1. Baseline Characteristics of the Study Participants

The characteristics of the 37 study participants are shown in Table 1. There were no statistically significant differences in age, sex, occupation, economic status, or lifestyle factors between the exposure and control groups (*p* > 0.05), suggesting that the groups were comparable at baseline. To biologically validate the heavy metal exposure status in our analytical subset, we compared available blood metal concentration data from individuals in the exposure(*n* = 15) to the historical control group (*n* = 16) from our cohort study [22]. As summarized in Table S1, the concentrations of Cd, Pb, and Zn were elevated in the exposure subset (*p* < 0.05), while the concentrations of copper Cu and Mo were not significantly different. This pattern is consistent with the established exposure profile of the broader Baiyin cohort.

### 3.2. Heavy Metal Stress Alters Oral Bacterial Composition in Residents

**Figure 1:**
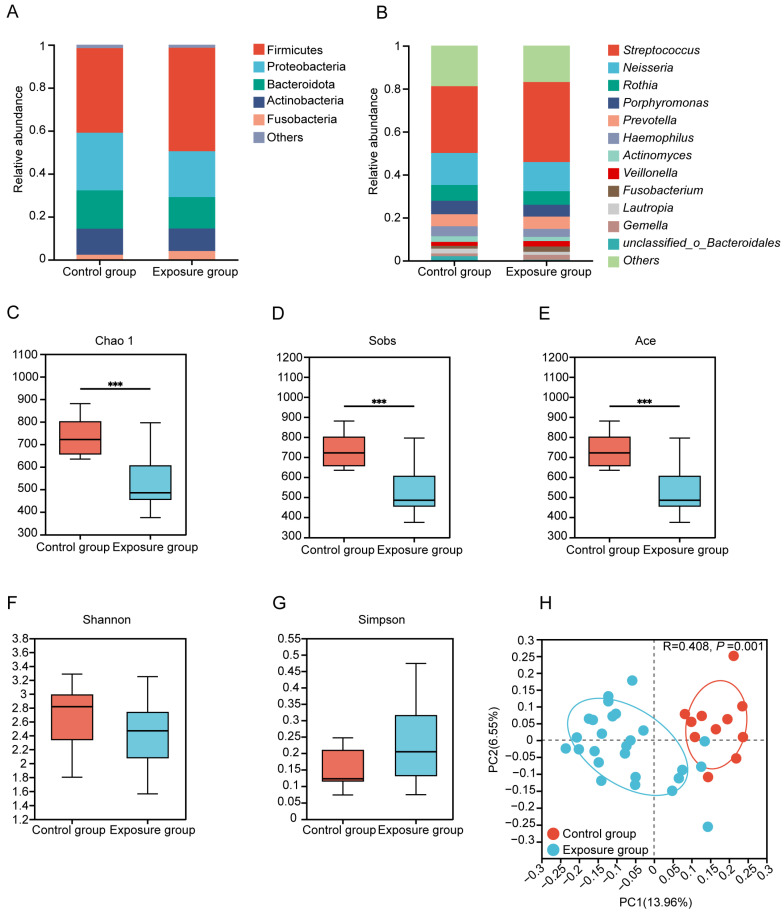
Composition and diversity of oral bacteria. (**A**) Bacterial relative abundance and composition at phylum and (**B**) genus levels. (**C**–**G**) Alpha diversity estimates of oral bacterial diversity and statistical significance (Wilcoxon test) between the exposure and control groups (*** *p* ≤ 0.001). (**H**) Binary Jaccard-based Principal coordinates analysis (PCoA) of the bacteria from the different groups.

Taxonomic profiling revealed there are five predominant (>2% abundance) phyla of oral microbiota in both groups, which are Firmicutes (48.14% vs. 39.39%), Proteobacteria (21.34% vs. 26.82%), Bacteroidota (14.65% vs. 17.82%), Actinobacteria (10.45% vs. 12.10%), and Fusobacteria (4.04% vs. 2.32%) (Figure 1A). Twelve dominant genera were observed in both groups, with the top three dominant genera being *Streptococcus* (exposure: 37.23% vs. control: 31.04%), *Neisseria* (13.57% vs. 14.92%), and *Rothia* (6.32% vs. 7.33%) (Figure 1B). Among these genera, *Faecalibacterium* demonstrated significantly greater abundance in exposure compared to control individuals (Figure S1). In addition, higher Chao1, Sobs, and ACE indices demonstrate significantly elevated bacterial richness in the control group (*p* < 0.001) (Figure 1C,D). However, metrics including the Shannon and Simpson indices showed no statistically significant intergroup differences (Figure 1F,G). Jaccard distance-based principal coordinate analysis (PCoA) demonstrated divergent community structures between the exposure and control groups (ANOSIM *R* = 0.408, *p* = 0.001) (Figure 1H).

Molecular ecological network analysis (MENA) was utilized to compare interactions within the human oral bacterial communities between groups. To ascertain whether the co-occurrence network differed from the randomized one, we utilized the Maslov-Sneppen program to generate a randomized version for comparison. The average clustering coefficient (avgCC) of both empirical networks was significantly higher than that of the randomized (Table S2), suggesting a nonrandomized pattern and small-world properties. The exposure group network consisted of 5 modules (with >5 nodes, module degree of 0.747), 119 nodes and 197 edges, whereas the control group network had 9 modules (module degree of 0.583), 595 nodes and 3000 edges (Figure S1; Table S3). The exposure group network exhibited fewer modules and reduced intra-module nodes and edges, demonstrating that heavy metal exposure interferes with human oral flora diversity and alters the connectivity patterns of human oral microbes. Among the network edges, positive correlations dominated microbial interactions (exposure: 95.94%; control: 66.97%), and the exposure group showed more cooperative relationships, which implied that heavy metal exposure enhanced synergistic interactions between human oral microorganisms.

### 3.3. Human Oral Resistome Profiles Under Heavy Metal Stress

**Figure 2:**
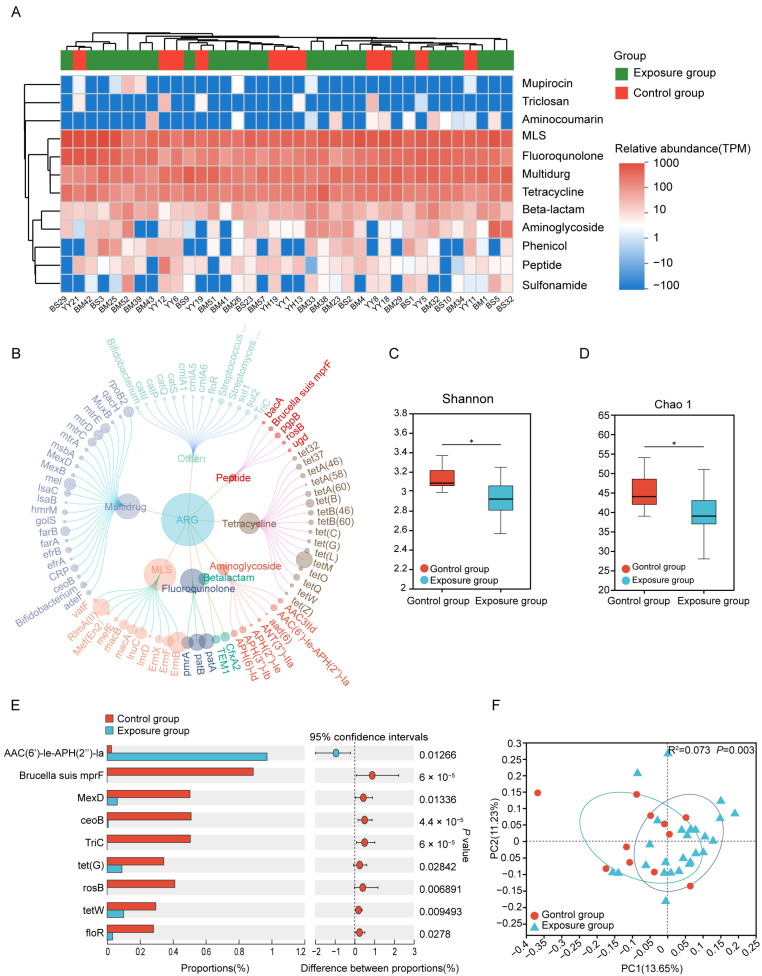
The composition and differences of ARGs between the exposure and control groups. (**A**) The classes of ARGs in different groups. (**B**) The antibiotic resistance subtypes of each class (the size of each node is proportional to the abundance). (**C**,**D**) Alpha diversity estimates of oral microbial ARGs diversity and statistical significance (Wilcoxon test) between the two groups. (**E**) Differences in the average relative abundance of ARGs(subtypes) between two groups (* *p* ≤ 0.05). (**F**) Binary Jaccard-based Principal coordinates analysis (PCoA) of the ARGs from the different groups. ARGs, antibiotic resistance genes.

Twelve types of ARGs were annotated in the oral samples of 37 residents by blasting against the CARD database (Figure 2A). The top five ARG types by relative abundance were macrolides-lincosamide-streptomycin, polypharmacy, fluoroquinolones, tetracyclines, and β-lactams. These 12 ARGs encompassed 81 ARG subtypes (Figure 2B), and we observed significantly altered diversity of resistance genes between the control and exposure groups (*p* < 0.05) (Figure 2C,D). Notably, the aminoglycoside-resistance gene *AAC(6′)-Ie-APH(2″)-Ia* was more abundant in the exposure group. In contrast, several other resistance genes were significantly enriched in the control group, including *Brucella suis mprF* (bacitracin resistance), *MexD* (multidrug resistance), *ceoB* (multidrug resistance), *TriC* (triclosan resistance), *tet(G)* (tetracycline resistance), *rosB* (peptide resistance), *tetW* (tetracycline resistance) and *floR* (phenicol resistance) (Figure 2E). Besides, some resistance genes displayed group specificity. Seven resistance genes were unique to the exposure group: aminoglycoside-resistance genes *aad(6)* and *APH(2″)-Ie*, phenicol-resistance genes *catP* and *cmlA5*, MLS-resistance gene *lmrD*, multidrug-resistance gene *lsaB*, and tetracycline-resistance gene *tetA(58)*. In contrast, the control group uniquely harbored the peptide-resistance gene *bacA*, *rosB* and *Brucella suis mprF*, the triclosan-resistance gene *TriC*, and the multidrug-resistance gene *golS* (Figure S2). Beyond these group-specific genes, the overall resistance gene composition, as visualized by the PCoA plot (Figure 2F), also differed significantly between exposure and control groups. This difference was statistically confirmed by Adonis analysis (*R*^2^ = 0.073, *p* = 0.003), suggesting that heavy metal exposure changes the human oral resistome profile. After adjusting for potential confounders (sex, age, smoking, drinking, occupation, income, and migrant status), heavy metal exposure remained a significant predictor of the ARG profile (Jaccard: *R*^2^ = 0.070, *p* < 0.001; Bray-Curtis: *R*^2^ = 0.0546, *p* < 0.05, Table S4). These results demonstrated heavy metal exposure has an independent effect on the oral resistome that is distinct from the other measured variables.

**Figure 3:**
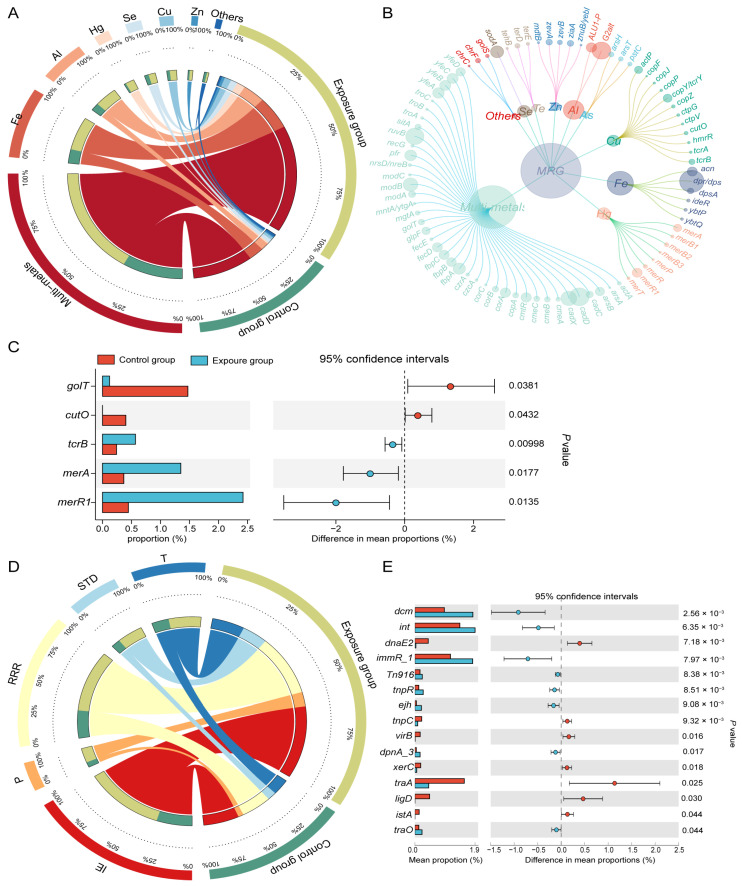
Composition and differences of MRGs and MGEs. (**A**) Distribution of the various types of MRGs (each bar length in the inner ring represents the percentage of MRG relative abundance) and (**B**) MRG subtypes (the size of each node is proportional to the abundance). (**C**) Differences in the average relative abundance of MRGs(subtypes) between two groups. (**D**) MGEs (MGE process) in exposure and control groups (each bar length in the inner ring represents the percentage of MGE relative abundance). (**E**) Differences in the average relative abundance of MGEs.

For MRGs, 83 subtypes were detected across 11 types through seraching the BacMet database (Figure 3A,B). Multimetal-related resistance genes predominated in both the exposure group (57.95%) and control group (63.09%), followed by resistance genes for iron (Fe) (17.83% vs. 15.75%) and aluminum (Al) (10.99% vs. 8.41%). Resistance genes associated with mercury (Hg), selenium (Se), copper (Cu), and zinc (Zn) were below 5% (Figure 3A). MRG alpha diversity showed the same patterns as ARG diversity between the two groups (Figure S2B,C). The Cu-resistant gene *tcrB* and the Hg-resistant genes *merA* and *merR* were significantly enriched in the exposed group (Figure 3C).

By blasting against the MGE database, 567 MGEs were identified and categorized into five groups based on their molecular mechanisms of mobilization (Figure 3D). Among these mechanisms, integration/excision (IE) was the most common, comprising 36.06% in the exposure group and 36.54% in the control group. Other prevalent mechanisms included replication/reorganization/repair (RRR) (30.43% vs. 31.09%), transfer processes (T) (14.69% vs. 17.52%), and stability/translocation/defense (STD) (11.70% vs. 9.32%). Phage-related MGEs (P) constituted the smallest proportion (7.13% vs. 5.53%). Certain MGEs, including the integrase-encoding gene int and the conjugated transposon *Tn916*, were significantly more abundant in the oral cavity of the heavy metal-exposed population than in the non-exposed population (Figure 3E).

### 3.4. Co-Selection Analysis of MRGs and ARGs

**Figure 4:**
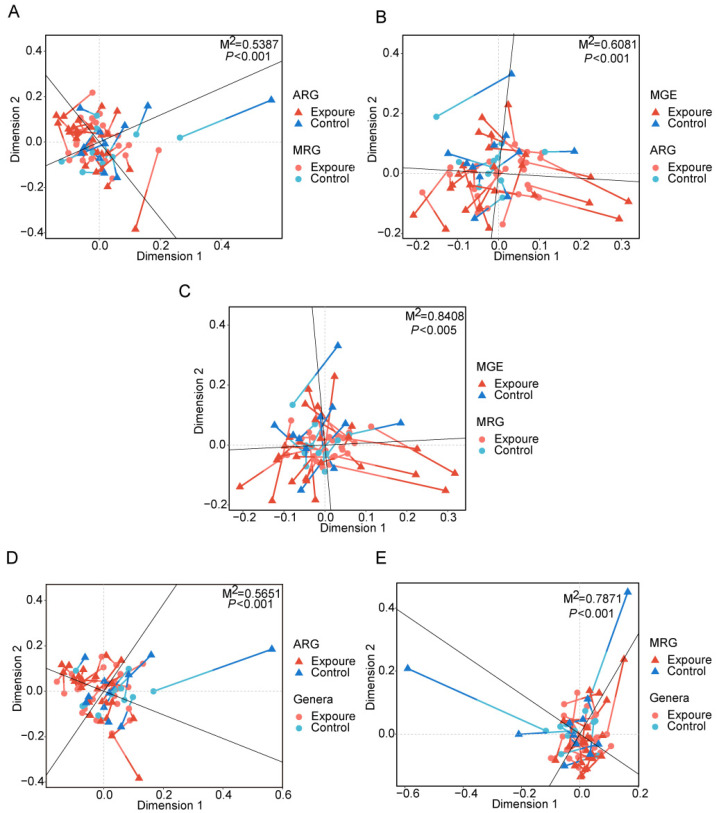
Procrustes analyses displaying significant correlations between (**A**) ARGs and MRGs, (**B**) ARGs and MGEs, (**C**) MRGs and MGEs, (**D**) ARGs and microbial community, (**E**) MRGs and microbial community. MRGs, metal resistance genes. MGEs, mobile genetic elements.

Procrustes analysis demonstrated significant concordance among the abundance profiles of ARGs, MRGs, and MGEs. Specifically, there was a strong association between ARGs and MRGs (*M*^2^ = 0.5387, *p* < 0.001, Figure 4A), ARGs and MGEs (*M*^2^ = 0.6081, *p* < 0.001, Figure 4B), and MRGs and MGEs (*M*^2^ = 0.8408, *p* < 0.005, Figure 4C). The lower *M^2^* value between ARGs and MGEs indicated MGEs imposed stronger selective pressure on ARGs than on MRGs in the human oral microbiome [47].

**Figure 5:**
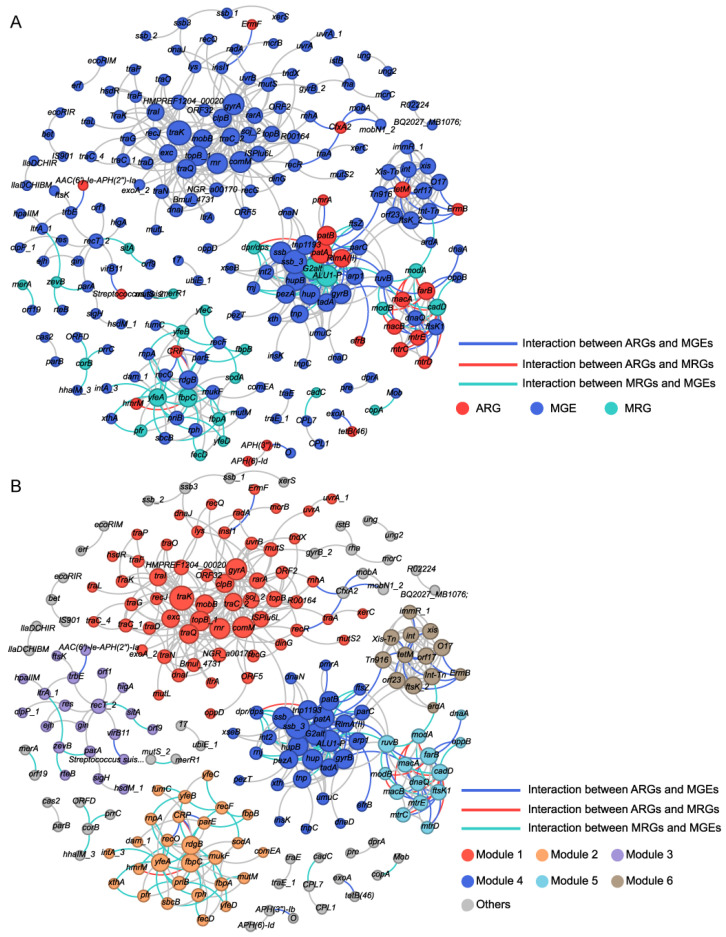
Co-occurrence patterns between MRGs, ARGs, and MGEs in all oral samples. (**A**) Each node was colored according to the MRGs, ARGs, and MGEs. (**B**) Each node was colored according to different modularity. Node size is proportional to the number of connections. Interactions of the same type are linked by gray lines.

A co-occurrence network was performed based on significant correlations among resistance genes (Figure 5A,B). Analysis revealed significant structural divergence between the observed microbial association network and corresponding random networks, confirming a non-random, highly connected topological structure (Tables S5 and S6). The observed network comprised 211 nodes (22 ARGs, 23 MRGs, 166 MGEs) forming 562 connections, with high modular organization (modularity index = 0.763), suggesting genes within the same module may exhibit synchronized co-occurrence under identical environmental stress [48]. Module 1 was most prominent (27.49% of nodes), followed sequentially by Module 4 (14.22%), Module 2 (12.80%), and Module 3 (9.95%) (Figure 5B). Strikingly, MRGs demonstrated greater involvement than ARGs in these network modules. For instance, Module 1 consisted of an MLS-related ARG (*ErmF*) and multiple MGEs, and Module 4 contained multiple ARGs (*RlmA(II)*, *efrB*, *patA*, *patB*, *pmrA*) and MRGs (*ALU1-P*, *G2alt*, *dpr/dps*), along with associated MGEs. Module 2 was primarily composed of MRGs and MGEs, with only two multidrug-related ARGs (*CRP*, *hmrM*). Module 3 contained two ARGs (*AAC(6′)-Ie-APH(2″)-Ia*, *Streptococcus suis chloramphenicol acetyltransferase*), two MRGs (*sitA*, *zevB*), and the rest were MGEs. Module 5 was dominated by six ARGs (*farB*, *macA*, *macB*, *mtrC*, *mtrD*, *mtrE*), with 3 MRGs and 5 MGEs. In the co-occurring network, most of the nodes were peripherals while *recT_2* (Module 3), *traK* (Module 1), *rdgB* (Module 2), and *fbpC* (Module 2) were module hubs, in which *fbpC* was MRG, and the remaining three were MGEs (Figure S3A). Among the 562 edges, 20 were between ARGs and MRGs (3.56%), all showing positive correlations. These included 13 multimetal-related MRGs, six Al-related MRGs, and one Fe-related MRG. In comparison, 77 edges (13.70%) connected ARGs and MGEs, with 70 (90.91%) showing positive connections. Additionally, 88 edges (15.66%) were between MRGs and MGEs, 74 of which (84.09%) were positively correlated. Overall, most MGEs showed stronger positive correlations with ARGs than with MRGs.

Potential co-occurrence patterns of MRGs, ARGs, and MGEs in each group were also evaluated (Figure S3B,C, Tables S7 and S8). Module 1 (MGE-dominated) showed a sharp decrease in node number in the exposed group, and the resistance gene *ermF* disappeared. Module 4, which contains multiple resistance genes, exhibited a reduced node count, though the Al resistance genes (*G2alt*, *ALU1-P*) and ARGs (*RlmA(II)*, *patA*, *patB*) persisted. Modules 2 and 3 lost most of their nodes. Module 5, which was ARG dominated, showed that MGEs were more strongly affected, with a decline in node number. In addition, compared to controls, the exposure group exhibited higher proportions of ARGs (11.49% vs. 10.05%) and MRGs (10.34% vs. 8.51%), but lower proportions of MGEs (78.16% vs. 81.44%). Furthermore, the exposure group showed stronger positive correlations between network nodes (97.46% vs. 58.17%). Meanwhile, the co-occurrence network of the exposure group had a lower average degree, suggesting that resistance genes in this group may be under stronger selective pressure [49].

### 3.5. Potential Bacterial Hosts for Resistance Genes

**Figure 6:**
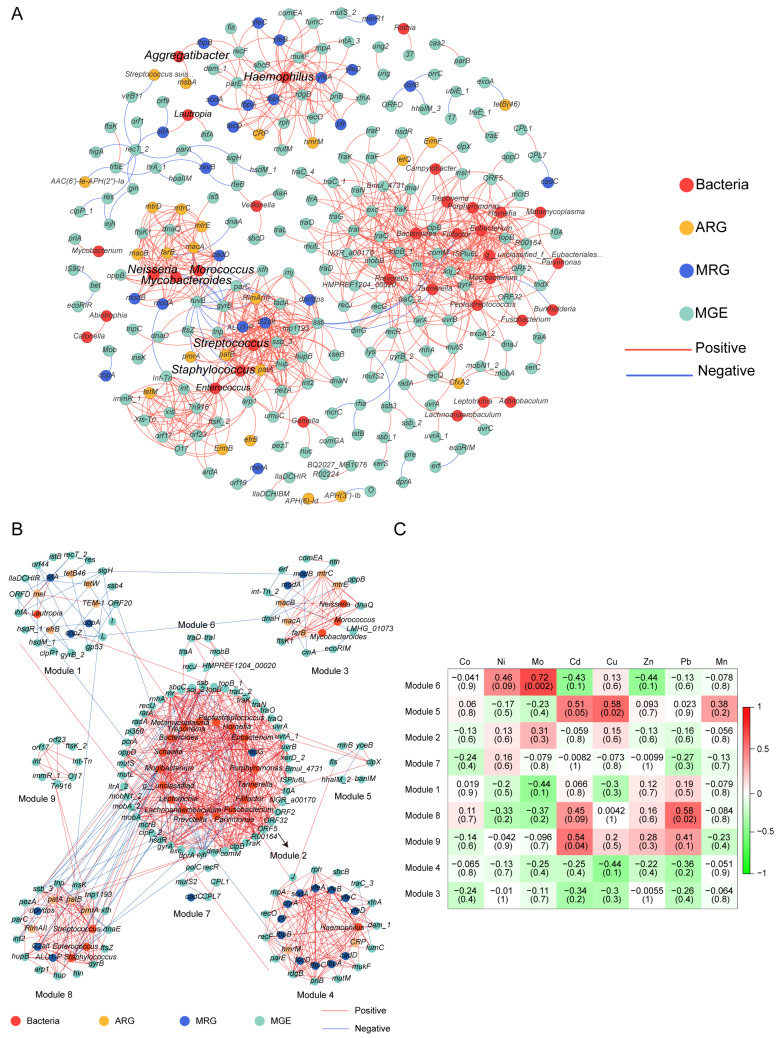
Network analysis shows MRGs, ARGs, and MGEs co-occurrence with microbial communities. (**A**) The co-occurrence patterns between the genes and bacteria in all oral samples. Each edge represents a strong and significant correlation (*r* > 0.65 (or <−0.65), *p* < 0.05). Nodes represent ARGs, MRGs, MGEs, and bacterial species at genera. (**B**) Coexistence network modules of genes and bacteria of oral samples from 15 subjects screened in the exposure group (determined by fast greedy module optimization method, only nodes greater than 5 are shown). (**C**) Correlation between blood metal levels and bacteria and genes in the module of 15 study subjects. Numbers represent correlation coefficients (*r*) and significance (*P*) is in parentheses.

**Figure 7:**
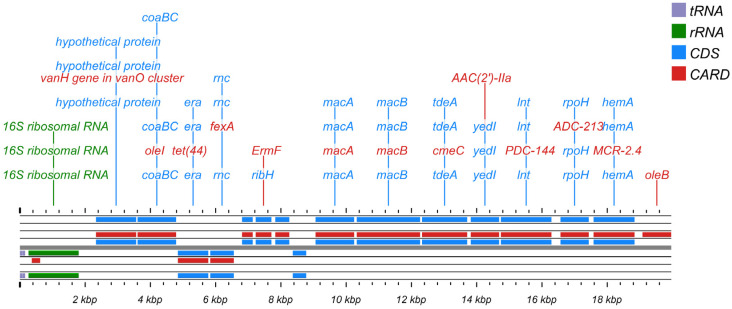
Functional annotation and structural characteristic map of the *Neisseria gonorrhoeae* gene sequence.

Procrustes analysis was conducted to evaluate the congruence between ARG distribution and microbial phylogenetic structure, using PCA ordinations of ARG subtype and bacterial species abundance matrices. Significant correlations between ARG profiles and microbial community composition (*M*^2^ = 0.5651, *p* < 0.001, Figure 4D) suggested the hypothesis that bacterial phylogeny influences ARG profiles in the human oral microbiome [50], and similar results were observed for MRGs (*M*^2^ = 0.7871, *p* < 0.001, Figure 4E). Procrustes analysis provided insight into the overall correlations between ARGs and the phylogenetic structure of microbial communities, but detailed information on ARG subtypes and microbe relationships was unknown. Therefore, we conducted network analysis to identify potential hosts taxa for MRGs, ARGs, and MGEs based on statistically positive correlations with bacterial genera (*r* > 0.65, *p* < 0.05, Figure 6A). Proteobacteria and Firmicutes emerged as the predominant potential hosts for carrying and transmitting MRGs and ARGs. Several bacterial genera, such as *Neisseria* and *Haemophilus* (Proteobacteria), *Morococcus*, *Streptococcus,* and *Staphylococcus* (Firmicutes), and *Mycobacteroides* (Actinobacteria) were related to various ARGs, MRGs, and MGEs. Notably, certain resistance genes appeared to share common bacterial hosts. For example, MRGs like *cadD* and *modA*, ARGs including *mtrD*, *mtrC*, *mtrE*, *macA*, *macB*, and *farB*, and MGEs such as *ftsK1*, *dnaQ*, and *ruvB* were all linked to *Neisseria*, *Morococcus*, and *Mycobacteroides*. Notably, a contig predicted to originate from *Neisseria gonorrhoeae* was found to harbor ARGs, MRG, and MGEs in proximity, suggesting a potential co-transferable genetic unit (Figure 7).

### 3.6. Human Blood Heavy Metals Validate Environment-Oral Bacterial Resistome Links

To more thoroughly assess the effect of heavy metals on human oral resistance genes, we analyzed data from subjects in the exposure group who had both blood samples and oral mucosa samples collected. The response patterns of the bacterial and resistance gene modules to heavy metal exposure were shown in the heatmap (Figure 6B,C). Several modules exhibited positive associations with blood heavy metal concentrations. Specifically, module 6 correlated with blood molybdenum (Mo), module 9 with blood cadmium (Cd), module 5 with blood copper (Cu), and module 8 correlated with blood lead (Pb). These metal-module correlation analyses were based on 15 subjects in the exposure group and 2 in the control group. Therefore, this analysis cannot robustly assess the statistical differences between the exposure group and the control group.

## 4. Discussion

Five dominant phyla in the human oral cavity were Firmicutes, Proteobacteria, Bacteroidetes, Actinobacteria, and Fusobacteria, aligning with established findings [51], indicating the reliability of the results. At the genus level, predominant were commensal genera (e.g., *Neisseria*, *Rossia*, *Haemophilus*, and *Actinobacteria*), along with some genera (e.g., *Streptococcus*, *Prevotella*, *Clostridium*, and *Veillonella*) linked to various health conditions [52]. Existing research has established that heavy metal exposure induces alterations in human oral microbiota composition [53]. Our microbiome analyses also demonstrated that prolonged exposure to heavy metals disrupted the human oral microbiota at the genus level. Individuals chronically exposed to heavy metals exhibited elevated relative abundances of *Faecalibacterium* in their oral microbiota (Figure S1), indicating adaptation to heavy metal stress.

Previous findings demonstrating environmental modulation of the human oral resistome [19], our study provided evidence that heavy metal exposure altered ARG diversity. We observed significantly reduced Shannon and Chao 1 indices in the exposure group versus controls (Figure 2C,D), suggesting the fluctuation of ARG diversity is related to metal exposure. Although overall alpha diversity decreased, the exposure group harbored more unique ARGs (Figure S2A). This discrepancy may be due to the appearance of certain specific ARGs in the exposure group. Existing research indicated that co-resistance occurred when an MRG and ARG were physically linked on the same genetic element, particularly on an MGE. Cross-resistance emerges when MRGs and ARGs are functionally related, typically through a single genetic element encoding a multifunctional detoxification system. Co-regulation occurs when these resistance genes are regulated by shared metal-responsive regulators or signaling pathways, indicating that metals serve as co-activators to induce MRG and ARG expression [54]. Thus, the increased presence of unique ARGs in the exposure group could be related to long-term heavy metal exposure, and metal-activated regulatory proteins may initiate the expression of ARGs. Alternatively, it was also possible that bacteria acquired new resistance genes through horizontal gene transfer [55]. Microorganisms adapt to new ecological niches under selective pressure, often gaining new resistance through HGT mediated by MGEs [56]. Based on network analysis results, the resistance gene *ermF* may have high transfer potential facilitated by MGEs, with its putative host Bacteroides, warranting particular attention.

Applying co-occurrence network analysis, we found ARGs exhibited stronger positive associations with MGEs compared to MRGs. This converged with the results from Procrustes analysis (Figure 4B,C), suggesting that human oral microbes may preferentially facilitate ARG dissemination through HGT in response to metalliferous stress. This contrasts with the driving model of MGEs on resistome observed in acid mine drainage of coal origin [47]. The contrast in this study may be attributed to both heavy metal exposure and residual agricultural antibiotics in soils. Direct exposure to antibiotics or the ingestion of ARGs via the food chain can affect the human resistance genome [57,58]. Furthermore, consistent with established patterns [59], microbial community structure was more important than the MGEs in driving the dynamics of the resistance group in this study (Figure 3B–E). Therefore, we hypothesized that environmental heavy metal exposure primarily influences changes in human oral ARGs through alterations in the human oral bacteria community. For example, the gene *AAC(6′)-le-APH(2″)-la* (resistance to aminoglycosides) (Figure 2E) exhibited significant enrichment in the exposure group, commonly found in both human and foodborne *Enterococcus* [60], and *Enterococcus* was capable of surviving in heavy metal-contaminated environments [61]. Network analyses also showed that some MRGs appeared alongside ARGs in the main module (Figure 5B). For example, the cadmium/zinc (Cd/Zn) resistance gene *cadD* was correlated with the *macA*, *macB*, *farB*, *mtrE*, *mtrC*, and *mtrD*. Previous studies have reported plasmid co-localization of *cadD*, a Cd/Zn resistance gene, with aminoglycoside and macrolide resistance genes [62]. This co-localization on MGEs supports the potential for heavy metal exposure to not only co-selects for antibiotic resistance but also facilitate HGT. Considering there is no direct evidence that *ALU1-P*, *G2alt*, *fbpC*, and *ARGs* were located on the same MGE, we speculated they might achieve co-resistance through other mechanisms mentioned above. Future studies should aim to elucidate the specific mechanisms through which environmental heavy metal exposure promotes and maintains bacterial resistance in the human oral microbiome.

From an ecological perspective, nodes occupying distinct topological roles based on established threshold values (*Z_i_* = 2.5, *P_i_* = 0.62) can be classified into four categories: peripherals (*Z_i_* < 2.5, *P_i_* ≤ 0.62), connectors (*Z_i_* < 2.5, *P_i_* > 0.62), modular hubs (*Z_i_* ≥ 2.5, *P_i_* ≤ 0.62), and network hubs (*Z_i_* ≥ 2.5, *P_i_* ≥ 0.62) [63]. Previous studies on ARG co-occurrence network analysis have concluded that hubs can serve as indicators of ARG abundance, allowing the assessment of co-occurring ARGs using a power function [64]. This suggested that future research could validate whether the four core genes identified in this study (Figure S3A) can represent other co-occurring genes within the module. Furthermore, our co-occurrence network analysis revealed distinct connectivity patterns between exposure and control groups in both bacterial and resistance gene networks (Figure S1B,C and Figure S3B,C). The networks of exposure group exhibited significantly simpler structures than those of the control group. The small-world nature of the bacterial network facilitated efficient system dynamics, allowing the effects of perturbations to be swiftly distributed throughout the network [65]. Positive correlations between nodes in network analyses typically indicate cooperative relationships, such as symbiosis, commensal interactions, and shared ecological needs [65]. In contrast, negative correlations signify competition for limited resources, the creation of artifacts, predation, and parasitism. The exposure group showed a higher ratio of positive to negative correlations within bacterial and resistance gene networks compared to the control group. We hypothesized that prolonged environmental heavy metals exposure may alters the relationships between microorganisms in the human oral cavity, whereby positive interactions among microorganisms might enhance their resistance to heavy metals [66]. Additionally, resistance genes appeared to collaborate to bolster microbial survival against environmental stresses. These results further imply that heavy metal exposure may disrupt human oral bacterial diversity.

Prior research has shown that non-random symbiosis patterns between resistance genes and bacterial taxa can predict potential hosts [48,64]. The observed correlations between MRGs and ARGs may be explained by either shared bacterial hosts harboring these resistance genes or by distinct host species maintaining co-resistance [67]. In this study, we found that certain MRGs (*cadD* and *modA*), ARGs (*mtrD*, *mtrC*, *mtrE*, *macA*, *macB*, and *farB*), and genes mediating the basic functions of bacterial MGEs (*ftsK1*, *dnaQ*, and *ruvB*) shared three potential host bacteria: *Neisseria*, *Morococcus*, and *Mycobacteroides* (Figure 6A). Previous studies have indicated that bacteria colonizing human ecological niches often harbor both ARGs and MRGs via plasmids, a phenomenon that may be closely linked to selective pressures within the host microenvironment. While this did not imply that metal selection was a major driver of human oral bacterial evolution (bacteria harboring both genes), it suggested that metal exposure can could potentially create a selective environment favoring bacteria with co-resistance mechanisms and enhancing the opportunity for HGT among these shared hosts [68]. For example, studies have reported that symbiotic *Neisseria* was an essential constituent of the human pharyngeal microbiome, and *Neisseria gonorrhoeae* may have obtained the *mtrCDE* efflux pump gene cluster from commensal *Neisseria*, contributing substantially to macrolide resistance in *N. gonorrhoeae* [69,70]. Given that our data link heavy metal exposure to the presence of both metal and antibiotic resistance determinants in potential host genera, we hypothesize that environmental heavy metal exposure might increase the risk of this process. However, these potential hosts require further validation by macrogenomics assembly and binning [71]. Overall, network analysis disclosed bacterial genus and resistance gene co-occurrence patterns, providing targets for resistance gene exploration. In addition, we detected significant relationships between certain co-occurrence network modules and heavy metal elements (Figure 6B,C). Pb exposure showed a strong positive association with a network module with fluoro-quinolone resistance genes and harboring potential hosts such as Streptococcus and Staphylococcus. Studies have found that Pb exposure can reduce the concentration of salivary agglutinin (SAG), thereby weakening oral immunity [72]. The weakening of this primary defense ability of the host can alter the ecological environment of the oral cavity, possibly providing growth advantages for bacterial groups that are more tolerant to such changes or more prone to reproduction under weakened immune surveillance. Therefore, the enrichment of lead-related modules may not only be the result of direct metal selection but also an indirect consequence of the impaired salivary antibacterial activity induced by Pb.

In contrast, Mo, Cd, and Cu were significantly associated with MGEs. This finding is particularly intriguing because, in contrast to Pb and Cd, the systemic blood concentrations of Cu and Mo were not significantly elevated in the exposure subset. The apparent disconnection between systemic metal concentrations and their association with the oral resistome can be explained by several factors. The localized exposure of the oral mucosa via inhalation and ingestion is likely elevated relative to systemic levels. The oral mucosa acts as a primary barrier and deposition site for ingested compounds, meaning the local microbiome may be exposed to a more concentrated dose than what is reflected in blood biomarkers [73]. Thus, the oral microbiome may experience a direct selective pressure that is not fully captured by blood measurements. Because MRGs are frequently co-localized with ARGs on MGEs, even sublethal levels of heavy metals are sufficient to select for multidrug resistance plasmids [74]. Resistance genes often impose minimal fitness costs on their bacterial hosts and can persist stably in microbial communities long after the initial selective pressure is reduced [75]. Therefore, the resistome may act as a legacy of past exposure. These suggest that a focus solely on systemic metal concentrations may underestimate the true biological effects of environmental exposure on the human microbiome. We speculate that microbial communities respond to subtle, sub-clinical environmental cues that traditional toxicological biomarkers can miss. Although this exploratory analysis has limitations, its results reveal the potential value of blood biomarkers in future research. To further delve into this, it is necessary to conduct research with a larger sample size and a more balanced design. Combining oral microbiome and resistance group data with detailed metal exposure data from whole blood will help clarify the impact of heavy metals on antimicrobial resistance.

This study has several limitations. Although the comparison of blood concentrations between the current exposure group and the historical control baseline indicates that the exposure gradient still exists, the sample size of the current control subgroup is relatively small. The imbalance between the exposure group and the control group increased the risk of false negatives and may limit the extrapolation of research results to a wider population. In the future, these preliminary results need to be further verified and expanded through increasing the sample size, prospective recruitment and balanced group design. Oral hygiene and dietary data were not systematically collected, potentially introducing residual confounding. Standardized assessments of these factors are needed in future studies. Metagenome-assembled genomes (MAGs) were not constructed to validate gene co-localization, leaving this for future confirmation with a more complete genomic context.

## 5. Conclusions

Our study reveals significant associations between heavy metal exposure and alterations in the compositional structure, biodiversity, and interspecies interactions of oral microbiota and ARGs, with bacterial community shifts identified as the predominant factor linked to ARG variation. The observed patterns support the potential importance of co-selection in sustaining resistance, and *recT_2*, *traK*, *rdgB*, and *fbpC* are proposed as indicator genes for monitoring oral ARGs in populations. Through comprehensive analysis, we have identified *Neisseria*, *Haemophilus*, *Morococcus*, *Streptococcus*, *Staphylococcus,* and *Mycobacteroides* as potential hosts for human oral resistance genes.

## Figures and Tables

**Table 1:** Baseline characteristics of the study participants.

	Exposure Group (*n* = 26) (%)	Control Group (*n* = 11) (%)	*p* Value
Age, years (mean ± SD)	56.46 ± 1.099	54.10 ± 1.595	0.253
Female	16 (61.5)	5 (45.5)	0.475
Occupation			0.844
Farmer	22 (84.6)	9 (82.8)	
Worker	1 (3.8)	1 (9.1)	
Self-employed individuals	1 (3.8)	0 (0.0)	
Others	2 (7.7)	1 (9.1)	
Long-term migrant workers	5 (19.2)	1 (10.0)	0.655
Smoking	5 (19.2)	5 (50.0)	0.100
Drinking	3 (11.5)	3 (30.0)	0.317
Annual household income			0.565
<CNY5000	0 (0.0)	1 (9.1)	
CNY5000 to CNY9000	6 (23.1)	2 (18.2)	
CNY10,000 to CNY29,999	13 (50.0)	6 (54.5)	
CNY30,000 to CNY49,999	3 (11.5)	1 (9.1)	
≥CNY50,000	3 (11.5)	0 (0.0)	
Not provided	1 (3.8)	1 (9.1)	

## Data Availability

The original contributions presented in this study can be found in online repositories. The raw sequence data have been deposited in the NCBI Sequence Read Archive under BioProject ID PRJNA1235640.

## Supplementary Materials

The following supporting information can be downloaded at: https://www.mdpi.com/article/10.3390/microorganisms13122814/s1. Figure S1: Bacterial communities and bacterial molecular ecological network at the genus level. (**A**) The difference in bacterial communities between exposure and control group (* *p*  <  0.05; ** *p*  <  0.01; *** *p*  <  0.001). (**B**) Exposure group bacterial network. (**C**) Control group bacterial network. Nodes represent taxa, as well as red and blue edges indicate positive and negative correlations, respectively; Figure S2: (**A**) The number of shared and unique ARGs (at the subtype level) between the control group and exposure, and the percentage of unique ARGs in each group. (**B,C**) Alpha diversity estimates of oral microbial MRGs diversity and statistical significance (Wilcoxon test) between the two groups; Figure S3: Co-occurrence network analysis showed (**A**) module-based topological roles of the detected ARGs, MRGs, and MEGs in all oral samples. Co-occurrence patterns among MRGs, ARGs, and MGEs in (**B**) exposure group and (**C**) control group. Nodes represent different kinds of genes, as well as red and blue edges indicate positive and negative correlations, respectively; Table S1: The concentration of metals in the blood of subjects living in both contaminated and control areas (unit: ng/mL); Table S2: Comparison of topological properties of MEN in the exposure and control groups with the corresponding randomized networks based on *t*-tests; Table S3: Topological properties of the empirical and 100 random MENs of microbial communities in the exposure and control groups; n.a. denotes no data available in the random algorithm; Table S4: PERMANOVA tests examining the effects of heavy metal exposure and covariates on oral microbial ARGs; Table S5: Topological properties of the empirical and 100 random co-occurrence networks of ARGs, MRGs, and MGEs in all oral samples; n.a. denotes no data available in the random algorithm; Table S6: Comparison of topological properties of co-occurrence networks of ARGs, MRGs, and MGEs in all oral samples with the corresponding randomized networks based on *t*-tests; Table S7: Topological properties of the empirical and 100 random Co-occurrence networks of ARGs, MRGs, and MGEs in the exposure and control groups; n.a. denotes no data available in the random algorithm; Table S8: Comparison of topological properties of co-occurrence networks of ARGs, MRGs, and MGEs in the exposure and control groups with the corresponding randomized networks based on *t*-tests; Table S9: Resistance gene co-localization (contig-level).

## Abbreviations

The following abbreviations are used in this manuscript:

ARG	antibiotic resistance gene
HGT	horizontal gene transfer
MRG	metal resistance gene
MGE	mobile genetic element
MENA	Molecular ecological network analysis
