# Supplementary material for: Effects of Long-Term Heavy Metal Exposure on Oral Microbial Antibiotic Resistance Genes of Residents in the Mining and Smelting Area
Source: Microorganisms. 2025 Dec 10;13(12):2814. doi: 10.3390/microorganisms13122814 (PMC12736263; doi:10.3390/microorganisms13122814)
Supplement: Supplementary file 1 [file microorganisms-13-02814-s001.zip › microorganisms-4006282-supplementary.pdf]

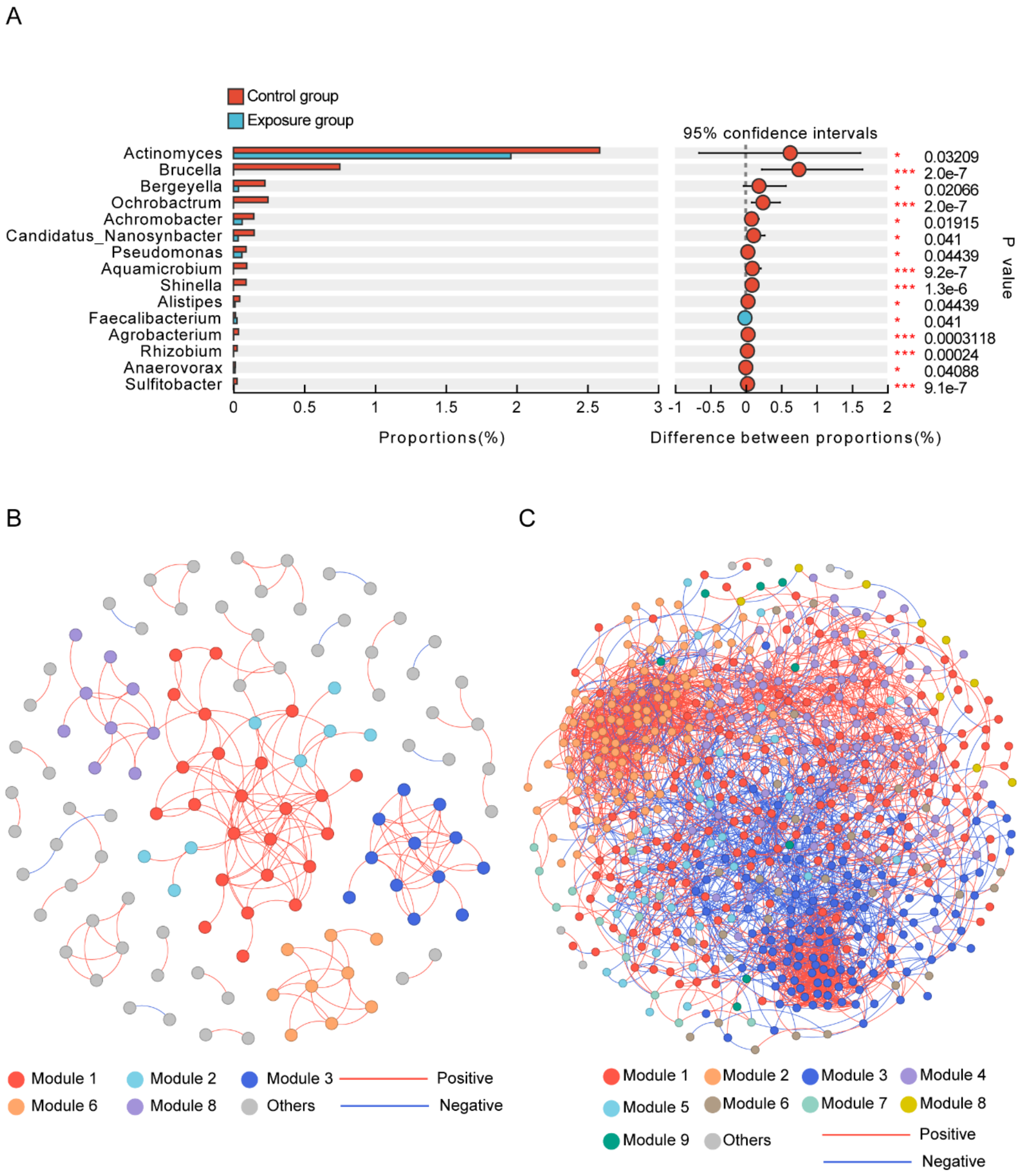

Figure S1 Bacterial communities and bacterial molecular ecological network at the genus level. **(A)** The difference in bacterial communities between exposure and control group (*$P < 0.05$; **$P < 0.01$; ***$P < 0.001$). **(B)** Exposure group bacterial network. **(C)** Control group bacterial network. Nodes represent taxa, as well as red and blue edges indicate positive and negative correlations, respectively.

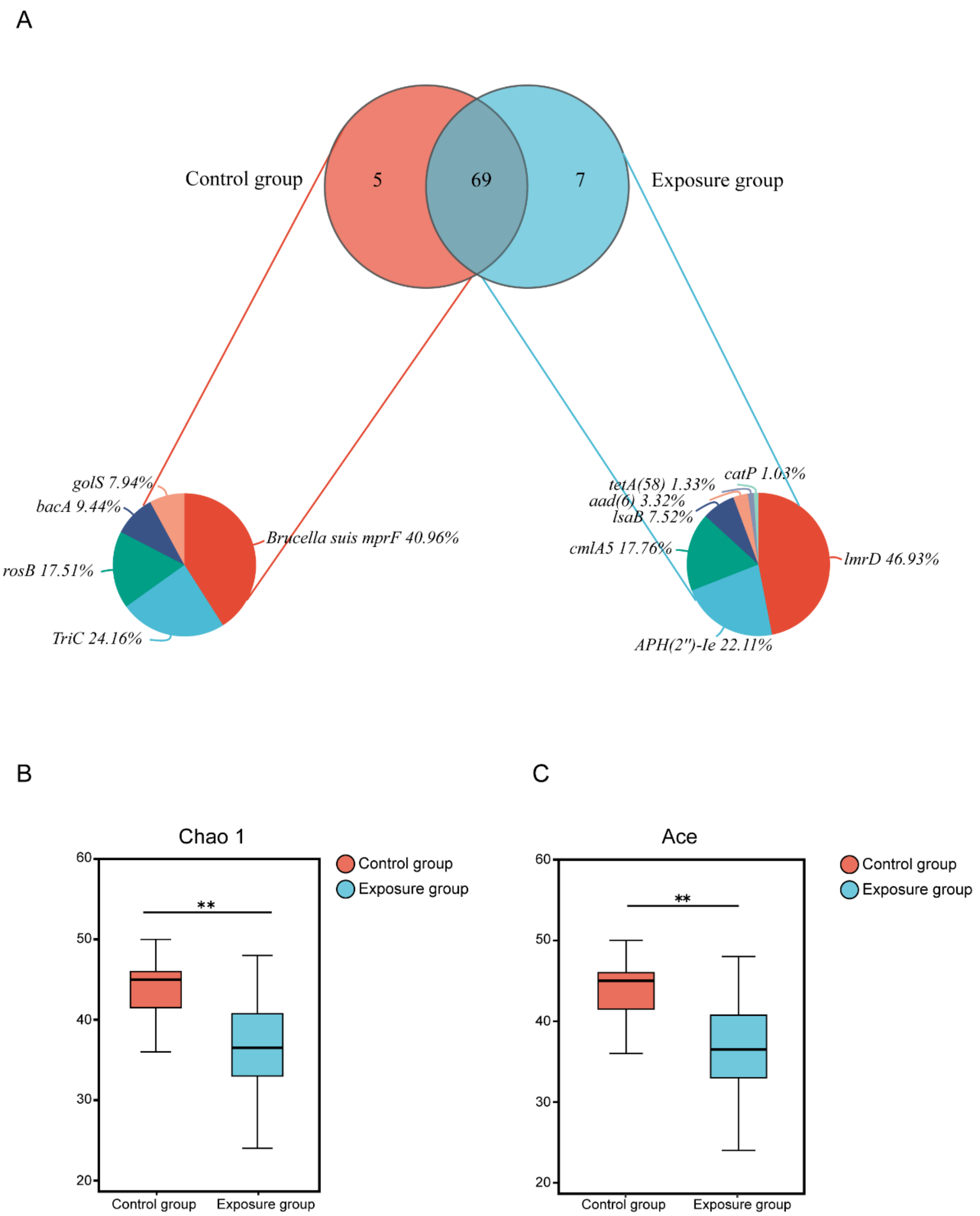

Figure S2 (A) The number of shared and unique ARGs (at the subtype level) between the control group and exposure, and the percentage of unique ARGs in each group. **(B, C)** Alpha diversity estimates of oral microbial MRGs diversity and statistical significance (Wilcoxon test) between the two groups.

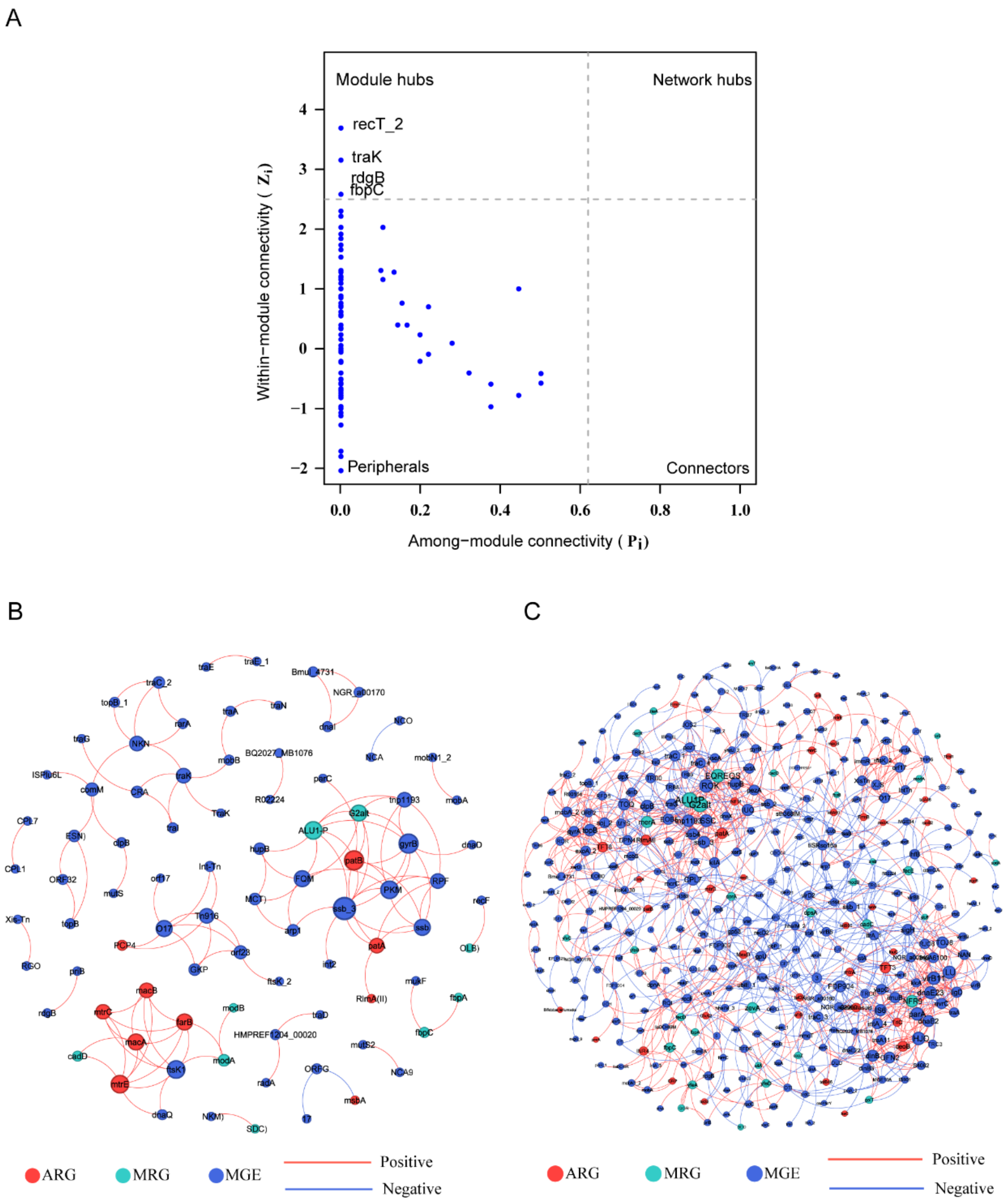

Figure S3 Co-occurrence network analysis showed **(A)**module-based topological roles of the detected ARGs, MRGs, and MEGs in all oral samples. Co-occurrence patterns among MRGs, ARGs, and MGEs in **(B)** exposure group and **(C)** control group. Nodes represent different kinds of genes, as well as red and blue edges indicate positive and negative correlations, respectively.

Table S1. The concentration of metals in the blood of subjects living in both contaminated and control areas (unit: ng/ml).

Element	Exposed Subset (n=15)	Historical Control Group (n=16) [22]	*P* Value
Co	0.60 ± 0.90	0.37 ± 0.04	<0.001
Ni	5.82 ± 8.40	4.89 ± 2.27	0.684
Mo	2.29 ± 1.49	2.15 ± 0.53	0.735
Cd	6.57 ± 2.77	1.91 ± 0.43	<0.001
Cu	863.38 ± 161.73	776.46 ± 34.43	0.059
Zn	5942.36 ± 948.46	5238.74 ± 196.88	0.013
Pb	54.78 ± 22.68	18.42 ± 1.13	<0.001
Mn	23.77 ± 17.47	15.62 ± 0.93	0.093

Table S2 Comparison of topological properties of MEN in the exposure and control groups with the corresponding randomized networks based on t-tests.

Index	avgCC		GD		Modularity	
Group	Exposure	Control	Exposure	Control	Exposure	Control
Empirical network	0.363	0.276	3.177	3.953	0.747	0.583
Random network	0.001±0.003	0.004±0.002	3.619±0.103	3.027±0.013	0.513±0.012	0.264±0.004
Student's *t*-value	1206.6667	1360	42.9126	712.3077	195	797.5
P-value	< 0.001	< 0.001	< 0.001	< 0.001	< 0.001	< 0.001

Table S3 Topological properties of the empirical and 100 random MENs of microbial communities in the exposure and control groups; n.a denotes no data available in the random algorithm.

Network indices	Exposure group	Control group	Exposure group	Control group
	Empirical		Random (mean ± SD)	
Total Nodes	119	595	n.a	n.a
Total links	197	3000	n.a	n.a
RMT cut-off	0.8	0.8	n.a	n.a
R square of power-law	0.978	0.666	n.a	n.a
Average degree(avgK)	3.311	10.084	n.a	n.a
Average clustering coefficient (avgCC)	0.363	0.276	0.001±0.003	0.004±0.002
Average path distance (GD)	3.177	3.953	3.619±0.103	3.027±0.013
Geodesic efficiency (E)	0.443	0.285	0.321±0.008	0.357±0.001
Harmonic geodesic distance (HD)	2.258	3.512	3.118±0.074	2.800±0.008
Centralization of degree (CD)	0.116	0.06	0.116±0	0.060±0
Centralization of betweenness (CB)	0.043	0.062	0.175±0.028	0.032±0.002
Centralization of stress centrality (CS)	0.196	0.599	0.051±0.027	0.136±0.022
Centralization of eigenvector centrality (CE)	0.923	0.936	0.851±0.016	0.819±0.009
Centralization of closeness centrality (CCL)	0.006	0.025	0.031±0.015	0.149±0.069
Density (D)	0.028	0.017	0.028±0	0.017±0
Reciprocity	1	1	1±0	1±0
Transitivity (Trans)	0.524	0.415	0.077±0.016	0.058±0.002
Connectedness (Con)	0.163	0.98	0.847±0.061	0.997±0.004
Efficiency	0.864	0.984	0.993±0.001	0.993±0
Hierarchy	0	0	0.028±0	0.017±0
Lubness	1	1	0.230±0.045	0.305±0.011
Modularity	0.747	0.583	0.513±0.012	0.264±0.004

Table S4 PERMANOVA tests examining the effects of heavy metal exposure and covariates on oral microbial ARGs

Variable	Jaccard Distance			Bray-Curtis Dissimilarity		
	R^2	*F* Value	*P* Value	R^2	*F* value	*P* Value
Group	0.06957	2.6679	0.0007***	0.05467	2.0113	0.0180*
Sex	0.0444	1.7026	0.0465*	0.04748	1.7468	0.0443*
Age	0.03034	1.1635	0.3077	0.01813	0.6671	0.8317
Occupation	0.11966	1.5296	0.0369*	0.10366	1.2712	0.1498
Smoking	0.05172	0.9916	0.4587	0.05628	1.0353	0.3707
Drinking	0.03602	1.3814	0.1525	0.03051	1.1226	0.3125
Income	0.09751	0.7479	0.9258	0.10997	0.8092	0.8341
Long-term migrant workers	0.02921	1.1199	0.3332	0.03569	1.3132	0.1711
Residual	0.52156			0.54361		
Total	1			1		

*P < 0.05, **P < 0.01, ***P < 0.001

Table S5 Topological properties of the empirical and 100 random co-occurrence networks of ARGs, MRGs, and MGEs in all oral samples; n.a denotes no data available in the random algorithm.

Network indices	Whole network	Random network
Total Nodes	211	n.a
Total links	562	n.a
RMT cut-off	0.65	n.a
R square of power-law	0.988	n.a
Average degree (avgK)	5.327	n.a
Average clustering coefficient (avgCC)	0.369	0.006±0.006
Average path distance (GD)	4.025	3.291±0.048
Geodesic efficiency (E)	0.327	0.341±0.004
Harmonic geodesic distance (HD)	3.062	2.929±0.032
Centralization of degree (CD)	0.084	0.084±0
Centralization of betweenness (CB)	0.183	0.073±0.01
Centralization of stress centrality (CS)	1.478	0.14±0.041
Centralization of eigenvector centrality (CE)	0.927	0.797±0.015
Centralization of closeness centrality (CCL)	0.005	0.036±0.04
Density (D)	0.025	0.025±0
Reciprocity	1	1±0
Transitivity (Trans)	0.534	0.085±0.006
Connectedness (Con)	0..382	0.935±0.03
Efficiency	0.944	0.991±0
Hierarchy	0	0.025±0
Lubness	1	0.347±0.028
Modularity	0.763	0.382±0.008

Table S6 Comparison of topological properties of co-occurrence networks of ARGs, MRGs, and MGEs in all oral samples with the corresponding randomized networks based on t-tests.

Indices	Whole network	Random network	Student's *t*-value	*P*-value
Average clustering coefficient (avgCC)	0.369	0.006±0.006	605	< 0.001
Average path distance (GD)	4.025	3.291±0.048	152.9167	< 0.001

Table S7 Topological properties of the empirical and 100 random Co-occurrence networks of ARGs, MRGs, and MGEs in the exposure and control groups; n.a denotes no data available in the random algorithm.

Network indices	Exposure group	Control group	Exposure group	Control group
	Empirical		Random(mean±SD)	
Total Nodes	87	388	n.a	n.a
Total links	118	1150	n.a	n.a
RMT cut-off	0.8	0.8	n.a	n.a
R square of power-law	0.961	0.75	n.a	n.a
Average degree(avgK)	2.713	5.928	n.a	n.a
Average clustering coefficient (avgCC)	0.322	0.243	0.002±0.007	0.003±0.003
Average path distance (GD)	2.439	4.704	3.892±0.174	3.507±0.021
Geodesic efficiency (E)	0.551	0.246	0.309±0.012	0.313±0.001
Harmonic geodesic distance (HD)	1.841	4.063	3.237±0.121	3.194±0.013
Centralization of degree (CD)	0.085	0.044	0.085±0	0.044±0
Centralization of betweenness (CB)	0.026	0.071	0.159±0.030	0.047±0.005
Centralization of stress centrality (CS)	0.025	0.452	0.031±0.019	0.110±0.026
Centralization of eigenvector centrality (CE)	0.91	0.946	0.812±0.027	0.811±0.017
Centralization of closeness centrality (CCL)	0.003	0.048	0.033±0.021	0.130±0.061
Density (D)	0.032	0.015	0.032±0	0.015±0
Reciprocity	1	1	1±0	1±0
Transitivity (Trans)	0.513	0.334	0.057±0.018	0.032±0.003
Connectedness (Con)	0.112	0.985	0.791±0.081	0.994±0.008
Efficiency	0.783	0.987	0.994±0.001	0.995±0
Hierarchy	0	0	0.032±0	0.015±0
Lubness	1	1	0.213±0.038	0.367±0.017
Modularity	0.793	0.626	0.578±0.016	0.381±0.006

Table S8 Comparison of topological properties of co-occurrence networks of ARGs, MRGs, and MGEs in the exposure and control groups with the corresponding randomized networks based on t-tests.

Index	avgCC		GD		Modularity	
Group	Exposure	Control	Exposure	Control	Exposure	Control
Empirical network	0.322	0.243	2.439	4.704	0.793	0.626
Random network	0.002±0.007	0.003±0.003	3.892±0.174	3.507±0.021	0.578±0.016	0.381±0.006
Student's *t* value	457.1429	800	83.5057	570	134.375	408.3333
P-value	< 0.001	< 0.001	< 0.001	< 0.001	< 0.001	< 0.001

Table S9 Resistance gene co-localization (contig-level)

Contig ID	Total Genes	Key Finding (Co-localization)	Gene Arrangement
YY8_k97_33951	5	2 ARGs + 1 MRG + 2 MGEs co-localized within a 9600 bp region.	MGE → ARG → ARG → MGE → MRG
B34_k97_100591	5	1 ARG + 1 MRG + 3 MGEs co-localized within a 31,105 bp genomic region.	MGE→MGE→ARG→MGE→MRG
B34_k97_107863	8	1 ARG + 1 MRG + 6 MGEs co-localized within a 29,738 bp genomic region.	MRG→MGE→MGE→MGE→MGE→MGE→ARG →MGE
B34_k97_109744	10	1 ARG + 2 MRGs + 7 MGEs co-localized within a 30,392 bp genomic region.	MGE→MGE→MGE→MGE→MGE→MGE→MGE →MRG→MRG→ARG
B34_k97_11488	14	5 ARGs + 1 MRG + 8 MGEs co-localized within a 65,247 bp genomic region.	MGE→MGE→MGE→ARG→MGE→ARG→ARG →MGE→MGE→MRG→MGE→MGE→ARG→AR G
B34_k97_115103	10	2 ARGs + 1 MRG + 7 MGEs co-localized within a 14,157 bp genomic region.	MGE→MGE→MGE→MGE→MGE→MGE→MGE →ARG→MRG→ARG
B34_k97_115408	13	2 ARGs + 1 MRG + 10 MGEs co-localized within a 36,289 bp genomic region.	MRG→MGE→MGE→MGE→MGE→MGE→MGE →MGE→MGE→ARG→MGE→ARG→MGE
B34_k97_120512	9	2 ARGs + 2 MRGs + 5 MGEs co-localized within a 16,923 bp genomic region.	MRG→MRG→MGE→MGE→MGE→MGE→ARG →ARG→MGE
B34_k97_122587	11	2 ARGs + 1 MRG + 8 MGEs co-localized within a 33,590 bp genomic region.	MGE→MGE→MGE→MGE→MGE→ARG→MGE →ARG→MGE→MGE→MRG
B34_k97_142731	14	1 ARG + 3 MRGs + 10 MGEs co-localized within a 47,179 bp genomic region.	MGE→ARG→MGE→MGE→MRG→MGE→MGE →MGE→MGE→MGE→MGE→MGE→MRG→M RG
B34_k97_151432	6	2 ARGs + 2 MRGs + 2 MGEs co-localized within a 18,148 bp genomic region.	ARG→MGE→MRG→MGE→ARG→MRG

B34_k97_154002	4	1 ARG + 1 MRG + 2 MGEs co-localized within a 4,554 bp genomic region.	ARG→MGE→MGE→MRG
B34_k97_27456	9	1 ARG + 1 MRG + 7 MGEs co-localized within a 33,607 bp genomic region.	MGE→MGE→MGE→MGE→MGE→MGE→MGE →ARG→MRG
B34_k97_37799	19	4 ARGs + 1 MRG + 14 MGEs co-localized within a 64,815 bp genomic region.	MGE→ARG→MGE→ARG→MRG→MGE→MGE →MGE→ARG→MGE→MGE→MGE→MGE→AR G→MGE→MGE→MGE→MGE→MGE
B34_k97_39560	14	1 ARG + 4 MRGs + 9 MGEs co-localized within a 58,793 bp genomic region.	MGE→MRG→MGE→MGE→MRG→MGE→MGE →MGE→MGE→MGE→MRG→ARG→MGE→MR G
B34_k97_39619	23	3 ARGs + 4 MRGs + 16 MGEs co-localized within a 54,890 bp genomic region.	MGE→MRG→MRG→ARG→MGE→MGE→MRG →MGE→MGE→MGE→MRG→MGE→MGE→AR G→MGE→MGE→ARG→MGE→MGE→MGE→M GE→MGE→MGE
B34_k97_48821	5	1 ARG + 1 MRG + 3 MGEs co-localized within a 16,047 bp genomic region.	ARG→MGE→MGE→MGE→MRG
B34_k97_59043	8	1 ARG + 1 MRG + 6 MGEs co-localized within a 47,020 bp genomic region.	MGE→MGE→MGE→MGE→MGE→MRG→ARG →MGE
B34_k97_68868	4	1 ARG + 1 MRG + 2 MGEs co-localized within a 3,542 bp genomic region.	MGE→MRG→MGE→ARG
B34_k97_77101	3	1 ARG + 1 MRG + 1 MGE in tight linkage within a 6,394 bp genomic region.	MGE→MRG→ARG
B34_k97_78405	6	1 ARG + 2 MRGs + 3 MGEs co-localized within a 19,109 bp genomic region.	MRG→ARG→MRG→MGE→MGE→MGE
B34_k97_92043	22	2 ARGs + 3 MRGs + 17 MGEs co-localized within a 59,885 bp genomic region.	MGE→MRG→MGE→MGE→MGE→MGE→MGE →ARG→MGE→MGE→MGE→MGE→MGE→MR

			G→MGE→MGE→MRG→MGE→MGE→ARG→MGE→MGE
B34_k97_92782	14	2 ARGs + 3 MRGs + 9 MGEs co-localized within a 42,800 bp genomic region.	MGE→MGE→MRG→MGE→MRG→MRG→ARG→MGE→MGE→MGE→MGE→MGE→MGE→ARG
BM25_k97_11808	3	1 ARG + 1 MRG + 1 MGE in tight linkage.	MGE→ARG→MRG
BM25_k97_12718	3	1 ARG + 1 MRG + 1 MGE in tight linkage.	MRG→MGE→ARG
BM25_k97_17718	3	1 ARG + 1 MRG + 1 MGE in tight linkage.	ARG→MGE→MRG
BM25_k97_17934	3	1 ARG + 1 MRG + 1 MGE in tight linkage.	MRG→ARG→MGE
BM25_k97_34837	7	2 ARGs + 2 MRGs + 3 MGEs in tight linkage, showing an alternating pattern.	ARG→MGE→MRG→ARG→MGE→MRG→MGE
BM25_k97_36105	3	1 ARG + 1 MRG + 1 MGE in tight linkage.	ARG→MRG→MGE
BM25_k97_38501	7	1 ARG + 1 MRG + 5 MGEs co-localized within a 16200 bp region.	MGE→MGE→ARG→MRG→MGE→MGE→MGE
BM25_k97_41410	4	1 ARG + 1 MRG + 2 MGEs in tight linkage.	MRG→MGE→ARG→MGE
BM25_k97_62025	20	2 ARGs + 1 MRG + 17 MGEs co-localized within a 57900 bp region. High gene density.	MGE→ARG→MGE→MGE→MGE→MRG→MGE... (20 genes total)
BM25_k97_64836	5	1 ARG + 1 MRG + 3 MGEs in tight linkage.	MRG→MGE→ARG→MGE→MGE
BM25_k97_65749	4	1 ARG + 2 MRGs + 1 MGE in tight linkage.	MGE→MRG→ARG→MRG
BM29_k97_12036	5	1 ARG + 2 MRGs + 2 MGEs in tight linkage, with very high gene density.	MRG→MGE→ARG→MGE→MRG
BM29_k97_12268	6	1 ARG + 1 MRG + 4 MGEs in tight linkage.	MGE→MGE→MGE→ARG→MGE→MRG
BM29_k97_13491	8	1 ARG + 1 MRG + 6 MGEs co-localized within a 27700 bp region.	MGE→MGE→MGE→MGE→MGE→MRG→ARG→MGE
BM29_k97_13915	8	1 ARG + 1 MRG + 6 MGEs in tight linkage.	MRG→MGE→MGE→ARG→MGE→MGE→MGE→MGE

BM29_k97_13944	7	2 ARGs + 1 MRG + 4 MGEs in tight linkage.	MGE→MGE→MRG→MGE→ARG→ARG→MGE
BM29_k97_15378	11	1 ARG + 1 MRG + 9 MGEs co-localized within a 23500 bp region.	ARG→MGE→MGE→MGE→MGE→MGE→MGE →MGE→MGE→MGE→MRG
BM29_k97_16575	3	1 ARG + 1 MRG + 1 MGE in tight linkage.	MRG→ARG→MGE
BM29_k97_16771	7	1 ARG + 2 MRGs + 4 MGEs in tight linkage.	MGE→MRG→MGE→MGE→ARG→MRG→MGE
BM29_k97_5684	4	1 ARG + 1 MRG + 2 MGEs in tight linkage.	ARG→MGE→MRG→MGE
BM29_k97_8296	10	1 ARG + 1 MRG + 8 MGEs co-localized within a 20200 bp region.	MGE→ARG→MGE→MGE→MRG→MGE→MGE →MGE→MGE→MGE
BM32_k97_10939	4	1 ARG + 2 MRGs + 1 MGE in tight linkage.	MGE → MRG → ARG → MRG
BM32_k97_12194	15	1 ARG + 2 MRGs + 12 MGEs co-localized within a 44500 bp region.	MRG → (MGE cluster) → ARG → MRG
BM32_k97_22779	3	1 ARG + 1 MRG + 1 MGE in tight linkage.	MGE → MRG → ARG
BM32_k97_29637	4	2 ARGs + 1 MRG + 1 MGE in tight linkage.	ARG → MGE → ARG → MRG
BM32_k97_6652	3	1 ARG + 1 MRG + 1 MGE in tight linkage within a 2300 bp region.	ARG → MRG → MGE
BM33_k97_102412	5	1 ARG + 1 MRG + 3 MGEs co-localized within a 16700 bp region.	MGE → MRG → ARG
BM33_k97_10939	4	1 ARG + 2 MRGs + 1 MGE in tight linkage.	MGE → MRG → ARG → MRG
BM33_k97_12194	15	1 ARG + 2 MRGs + 12 MGEs co-localized within a 44500 bp region.	MRG → (MGE cluster) → ARG → MRG
BM33_k97_126241	4	1 ARG + 1 MRG + 2 MGEs in tight linkage within a 12400 bp region.	MGE → ARG → MRG
BM33_k97_127001	5	1 ARG + 1 MRG + 3 MGEs co-localized within a 13800 bp region.	MGE → MRG → ARG
BM33_k97_140040	7	2 ARGs + 1 MRG + 4 MGEs co-localized within a 17000 bp region.	(MGE cluster) → ARG → MRG → ARG

BM33_k97_140787	12	2 ARGs + 2 MRGs + 8 MGEs co-localized within a 28300 bp region.	MGE → ARG → MRG → MRG → ARG
BM33_k97_143809	3	1 ARG + 1 MRG + 1 MGE in tight linkage within a 6040 bp region.	MRG → MGE → ARG
BM33_k97_150063	3	1 ARG + 1 MRG + 1 MGE in tight linkage within a 11400 bp region.	MGE → MRG → ARG
BM33_k97_150825	11	1 ARG + 2 MRGs + 8 MGEs co-localized within a 19200 bp region.	MGE → ARG → (MGE cluster) → MRG → MRG
BM33_k97_154048	6	1 ARG + 1 MRG + 4 MGEs co-localized within a 13400 bp region.	MGE → MRG → ARG
BM33_k97_162427	3	1 ARG + 1 MRG + 1 MGE in tight linkage within a 6970 bp region.	ARG → MRG → MGE
BM33_k97_16346	8	3 ARGs + 1 MRG + 4 MGEs co-localized within a 14400 bp region.	MGE → ARG → MRG → ARG → ARG
BM33_k97_175805	17	3 ARGs + 1 MRG + 13 MGEs co-localized within a 105100 bp region.	MGE → MRG → ARG → (MGE cluster) → ARG → ARG
BM33_k97_186170	4	1 ARG + 1 MRG + 2 MGEs in tight linkage within a 9730 bp region.	ARG → MGE → MRG
BM33_k97_188142	4	1 ARG + 1 MRG + 2 MGEs in tight linkage within a 23600 bp region.	(MGE cluster) → MRG → ARG
BM33_k97_192543	3	1 ARG + 1 MRG + 1 MGE in tight linkage within a 3060 bp region.	MRG → ARG → MGE
BM33_k97_196517	4	1 ARG + 1 MRG + 2 MGEs in tight linkage within a 12800 bp region.	MGE → ARG → MRG
BM33_k97_222708	13	3 ARGs + 1 MRG + 9 MGEs co-localized within a 36500 bp region.	MGE → MRG → ARG → ARG → ARG

BM33_k97_222949	7	1 ARG + 1 MRG + 5 MGEs co-localized within a 51000 bp region.	(MGE cluster) → ARG → MRG
BM33_k97_223412	10	2 ARGs + 2 MRGs + 6 MGEs co-localized within a 41500 bp region.	MRG → ARG → MRG → ARG
BM33_k97_22779	3	1 ARG + 1 MRG + 1 MGE in tight linkage.	MGE → MRG → ARG
BM33_k97_231282	7	2 ARGs + 2 MRGs + 3 MGEs co-localized within a 48100 bp region.	MRG → MGE → MRG → ARG → ARG
BM33_k97_231658	10	3 ARGs + 3 MRGs + 4 MGEs co-localized within a 41700 bp region.	MRG → ARG → MRG → ARG → ARG → MRG
BM33_k97_239228	3	1 ARG + 1 MRG + 1 MGE in tight linkage within a 18500 bp region.	MGE → MRG → ARG
BM33_k97_247599	8	1 ARG + 2 MRGs + 5 MGEs co-localized within a 34300 bp region.	(MGE cluster) → ARG → MRG → MRG
BM33_k97_25067	8	1 ARG + 1 MRG + 6 MGEs co-localized within a 19400 bp region.	(MGE cluster) → ARG → MRG
BM33_k97_259150	15	3 ARGs + 1 MRG + 11 MGEs co-localized within a 54200 bp region.	(MGE cluster) → ARG → ARG → ARG → MRG
BM33_k97_268616	3	1 ARG + 1 MRG + 1 MGE in tight linkage within a 4780 bp region.	MRG → MGE → ARG
BM33_k97_269462	5	3 ARGs + 1 MRG + 1 MGE in tight linkage within a 10200 bp region.	MRG → ARG → ARG → ARG → MGE
BM33_k97_29637	4	2 ARGs + 1 MRG + 1 MGE in tight linkage.	ARG → MGE → ARG → MRG
BM33_k97_306112	6	1 ARG + 1 MRG + 4 MGEs co-localized within a 17200 bp region.	MGE → ARG → MRG
BM33_k97_30631	3	1 ARG + 1 MRG + 1 MGE in tight linkage within a 12500 bp region.	MRG → ARG → MGE

BM33_k97_311386	3	1 ARG + 1 MRG + 1 MGE in tight linkage within a 5970 bp region.	ARG → MGE → MRG
BM33_k97_317831	3	1 ARG + 1 MRG + 1 MGE in tight linkage within a 6570 bp region.	ARG → MGE → MRG
BM33_k97_31994	3	1 ARG + 1 MRG + 1 MGE in tight linkage within a 11700 bp region.	ARG → MGE → MRG
BM33_k97_328264	14	2 ARGs + 1 MRG + 11 MGEs co-localized within a 41200 bp region.	(MGE cluster) → ARG → MRG → ARG
BM33_k97_33629	3	1 ARG + 1 MRG + 1 MGE in tight linkage within a 14100 bp region.	MGE → ARG → MRG
BM33_k97_33673	16	1 ARG + 5 MRGs + 10 MGEs co-localized within a 73100 bp region.	MRG → MRG → MRG → ARG → (MGE cluster) → MRG
BM33_k97_341753	10	2 ARGs + 1 MRG + 7 MGEs co-localized within a 15300 bp region.	(MGE cluster) → ARG → MRG → ARG
BM33_k97_341826	18	2 ARGs + 1 MRG + 15 MGEs co-localized within a 45900 bp region.	(MGE cluster) → ARG → ARG → MRG
BM33_k97_348103	3	1 ARG + 1 MRG + 1 MGE in tight linkage within a 4120 bp region.	MGE → MRG → ARG
BM33_k97_350681	5	1 ARG + 2 MRGs + 2 MGEs in tight linkage within a 6640 bp region.	MRG → MRG → ARG → MGE → MGE
BM33_k97_350771	8	1 ARG + 1 MRG + 6 MGEs co-localized within a 13400 bb region.	MGE → ARG → MRG
BM33_k97_362719	16	4 ARGs + 3 MRGs + 9 MGEs co-localized within an 80000 bp region.	MRG → (MGE cluster) → ARG → MRG → ARG → ARG → MRG → ARG
BM33_k97_373347	4	1 ARG + 2 MRGs + 1 MGE in tight linkage within a 9560 bp region.	MRG → ARG → MRG → MGE

BM33_k97_380722	3	1 ARG + 1 MRG + 1 MGE in tight linkage within a 48400 bp region.	MGE → MRG → ARG
BM33_k97_387830	3	1 ARG + 1 MRG + 1 MGE in tight linkage within a 3330 bp region.	MRG → MGE → ARG
BM33_k97_389882	5	1 ARG + 1 MRG + 3 MGEs co-localized within a 13800 bp region.	(MGE cluster) → MRG → ARG
BM33_k97_395631	5	1 ARG + 2 MRGs + 2 MGEs in tight linkage within a 7990 bp region.	MRG → MRG → ARG → MGE → MGE
BM33_k97_39707	4	1 ARG + 1 MRG + 2 MGEs in tight linkage within a 9950 bp region.	MRG → MGE → ARG
BM33_k97_400327	3	1 ARG + 1 MRG + 1 MGE in tight linkage within a 5100 bp region.	ARG → MGE → MRG
BM33_k97_402214	4	1 ARG + 2 MRGs + 1 MGE in tight linkage within a 6660 bp region.	MGE → MRG → ARG → MRG
BM33_k97_404197	5	1 ARG + 1 MRG + 3 MGEs co-localized within a 27600 bp region.	(MGE cluster) → MRG → ARG
BM33_k97_409093	13	2 ARGs + 1 MRG + 10 MGEs co-localized within an 82700 bp region.	ARG → (MGE cluster) → ARG → MRG
BM33_k97_421534	4	1 ARG + 1 MRG + 2 MGEs in tight linkage within a 6710 bp region.	MRG → MGE → ARG
BM33_k97_449766	5	1 ARG + 1 MRG + 3 MGEs co-localized within a 10300 bp region.	MGE → MRG → ARG
BM33_k97_452959	4	1 ARG + 1 MRG + 2 MGEs in tight linkage within a 4480 bp region.	MGE → MRG → ARG
BM33_k97_51424	4	1 ARG + 1 MRG + 2 MGEs in tight linkage within a 6850 bp region.	(MGE cluster) → MRG → ARG

BM33_k97_54804	4	1 ARG + 1 MRG + 2 MGEs in tight linkage within a 7140 bp region.	ARG → MRG → MGE → MGE
BM33_k97_60467	21	1 ARG + 4 MRGs + 16 MGEs co-localized within a 61400 bp region.	MGE → MGE → MGE → MRG → (MGE cluster) → ARG → (MGE/MRG cluster)
BM33_k97_6652	3	1 ARG + 1 MRG + 1 MGE in tight linkage within a 2300 bp region.	ARG → MRG → MGE
BM33_k97_73216	21	1 ARG + 2 MRGs + 18 MGEs co-localized within a 48600 bp region.	MGE → MGE → MRG → MGE → MRG → MGE → ARG → (MGE cluster)
BM33_k97_76925	6	1 ARG + 1 MRG + 4 MGEs co-localized within a 31600 bp region.	MGE → MRG → ARG
BM33_k97_87092	3	1 ARG + 1 MRG + 1 MGE in tight linkage within a 10000 bp region.	MRG → ARG → MGE
BM33_k97_95049	4	1 ARG + 1 MRG + 2 MGEs in tight linkage within a 9800 bp region.	MRG → MGE → ARG
BM33_k97_9953	10	2 ARGs + 2 MRGs + 6 MGEs co-localized within a 60000 bp region.	MGE → ARG → ARG → MRG → MRG
BM39_k97_86108	9	1 ARG + 1 MRG + 7 MGEs co-localized within a 14,998 bp genomic region.	MRG→MGE→MGE→MGE→MGE→MGE→MGE →ARG→MGE
BM42_k97_9475	3	1 ARG + 1 MRG + 1 MGE in tight linkage within a 3,576 bp genomic region.	ARG→MRG→MGE
BM51_k97_3986	4	1 ARG + 1 MRG + 2 MGEs in tight linkage within a 10,248 bp genomic region.	MGE→ARG→MGE→MRG
BM51_k97_4815	3	1 ARG + 1 MRG + 1 MGE in tight linkage within a 7,193 bp genomic region.	ARG→MGE→MRG
BM51_k97_6434	7	2 ARGs + 2 MRGs + 3 MGEs co-localized within a 7,755 bp genomic region.	MGE→MGE→MGE→ARG→MRG→MRG→ARG

BM51_k97_7841	3	1 ARG + 1 MRG + 1 MGE in tight linkage within a 4,995 bp genomic region.	MGE→MRG→ARG
BM51_k97_9941	4	2 ARGs + 1 MRG + 1 MGE in tight linkage within a 2,645 bp genomic region.	ARG→MGE→ARG→MRG
BM57_k97_3462	4	2 ARGs + 1 MRG + 1 MGE in tight linkage within a 2,646 bp genomic region.	MRG→ARG→MGE→ARG
BS1_k97_12765	25	1 ARG + 4 MRGs + 20 MGEs co-localized within a 173,998 bp genomic region.	MGE→MGE→MGE→ARG→MGE→MGE→MGE→MGE→MRG→MGE→MGE→MRG→MRG→MGE→MGE→MGE→MRG→MGE→MGE→MGE→MGE→MGE→MGE→MGE→MGE
BS1_k97_17942	19	5 ARGs + 1 MRG + 13 MGEs co-localized within a 83,488 bp genomic region.	MGE→MGE→MGE→MGE→MRG→MGE→MGE→ARG→ARG→MGE→ARG→ARG→ARG→MGE→MGE→MGE→MGE→MGE→MGE
BS1_k97_21334	19	1 ARG + 4 MRGs + 14 MGEs co-localized within a 96,740 bp genomic region.	MRG→MGE→MGE→MGE→MGE→MGE→MGE→MGE→MGE→MGE→MGE→MGE→ARG→MGE→MGE→MRG→MGE→MRG→MRG
BS1_k97_24991	11	3 ARGs + 1 MRG + 7 MGEs in tight linkage within a 75,172 bp genomic region.	ARG→MGE→ARG→MGE→MGE→MGE→ARG→MRG→MGE→MGE→MGE
BS1_k97_26510	5	1 ARG + 1 MRG + 3 MGEs in tight linkage within a 16,940 bp genomic region.	ARG→MGE→MRG→MGE→MGE
BS1_k97_28634	10	1 ARG + 2 MRGs + 7 MGEs in tight linkage within a 54,369 bp genomic region.	MRG→MGE→MGE→MGE→MGE→MGE→MGE→MGE→MRG→ARG
BS1_k97_29190	7	1 ARG + 2 MRGs + 4 MGEs in tight linkage within a 18,299 bp genomic region.	MGE→MRG→ARG→MRG→MGE→MGE→MGE
BS1_k97_3337	3	1 ARG + 1 MRG + 1 MGE in tight linkage within a 10,408 bp genomic region.	ARG→MRG→MGE

BS1_k97_36242	7	1 ARG + 1 MRG + 5 MGEs in tight linkage within a 9,976 bp genomic region.	MGE→MGE→MGE→MRG→MGE→ARG→MGE
BS1_k97_3913	15	2 ARGs + 2 MRGs + 11 MGEs co-localized within a 51,032 bp genomic region.	MGE→ARG→MGE→MGE→ARG→MGE→MGE→MGE→MGE→MGE→MGE→MRG→MRG→MGE→MGE
BS1_k97_39960	15	1 ARG + 2 MRGs + 12 MGEs co-localized within a 65,451 bp genomic region.	MGE→MGE→MGE→MGE→MGE→MRG→MGE→MGE→ARG→MGE→MGE→MGE→MGE→MRG→MGE
BS1_k97_40974	4	1 ARG + 1 MRG + 2 MGEs in tight linkage within a 10,254 bp genomic region.	MGE→MRG→MGE→ARG
BS1_k97_40995	15	1 ARG + 1 MRG + 13 MGEs co-localized within a 55,474 bp genomic region.	MGE→MGE→MGE→ARG→MGE→MGE→MGE→MGE→MGE→MRG→MGE→MGE→MGE→MGE→MGE
BS1_k97_41418	4	2 ARGs + 1 MRG + 1 MGE in tight linkage within a 7,596 bp genomic region.	ARG→MGE→ARG→MRG
BS1_k97_44969	15	4 ARGs + 3 MRGs + 8 MGEs co-localized within a 54,797 bp genomic region.	MRG→MRG→MRG→MGE→MGE→MGE→MGE→MGE→MGE→ARG→MGE→MGE→ARG→ARG→ARG
BS1_k97_56176	5	1 ARG + 1 MRG + 3 MGEs in tight linkage within a 20,554 bp genomic region.	MGE→MGE→ARG→MGE→MRG
BS1_k97_61628	4	1 ARG + 1 MRG + 2 MGEs in tight linkage within a 9,192 bp genomic region.	MGE→MGE→ARG→MRG
BS1_k97_65905	7	1 ARG + 1 MRG + 5 MGEs in tight linkage within a 23,426 bp genomic region.	MGE→MGE→MGE→MGE→MRG→ARG→MGE
BS1_k97_69513	13	2 ARGs + 1 MRG + 10 MGEs co-localized within a 39,904 bp genomic region.	MGE→MGE→MGE→ARG→ARG→MRG→MGE→MGE→MGE→MGE→MGE→MGE→MGE

BS1_k97_71095	33	3 ARGs + 1 MRG + 29 MGEs co-localized within a 99,567 bp genomic region.	ARG→MGE→MGE→MGE→MGE→MGE→MGE→MGE→MGE→MGE→MGE→MGE→MGE→MGE→MGE→MGE→MGE→MRG→MGE→MGE→MGE→MGE→ARG→ARG→MGE→MGE→MGE→MGE→MGE→MGE→MGE→MGE→MGE
BS1_k97_72909	14	1 ARG + 2 MRGs + 11 MGEs co-localized within a 44,240 bp genomic region.	MGE→MGE→MGE→ARG→MGE→MGE→MGE→MGE→MGE→MGE→MRG→MRG→MGE→MGE
BS1_k97_73853	9	1 ARG + 2 MRGs + 6 MGEs co-localized within a 26,866 bp genomic region.	MGE→MGE→MGE→MGE→MGE→MRG→MRG→MGE→ARG
BS10_k97_18660	12	1 ARG + 1 MRG + 10 MGEs co-localized within a 37,104 bp genomic region.	MGE→MGE→MGE→ARG→MGE→MGE→MGE→MGE→MGE→MGE→MRG→MGE
BS10_k97_19011	13	4 ARGs + 1 MRG + 8 MGEs co-localized within a 39,442 bp genomic region.	MGE→MGE→MRG→MGE→ARG→ARG→MGE→MGE→MGE→MGE→ARG→ARG→MGE
BS10_k97_31201	7	1 ARG + 1 MRG + 5 MGEs in tight linkage within a 17,201 bp genomic region.	MGE→MGE→MGE→MRG→ARG→MGE→MGE
BS10_k97_42145	11	1 ARG + 2 MRGs + 8 MGEs co-localized within a 34,111 bp genomic region.	MGE→MGE→MGE→MGE→MGE→MRG→ARG→MRG→MGE→MGE→MGE
BS10_k97_43621	6	2 ARGs + 1 MRG + 3 MGEs in tight linkage within a 14,511 bp genomic region.	MGE→ARG→ARG→MGE→MRG→MGE
BS10_k97_58654	5	1 ARG + 1 MRG + 3 MGEs in tight linkage within a 8,568 bp genomic region.	MRG→MGE→ARG→MGE→MGE
BS10_k97_61106	10	1 ARG + 2 MRGs + 7 MGEs co-localized within a 17,567 bp genomic region.	MRG→MRG→MGE→MGE→MGE→MGE→MGE→MGE→ARG→MGE
BS10_k97_64127	9	1 ARG + 1 MRG + 7 MGEs co-localized within a 18,551 bp genomic region.	MGE→MGE→MGE→MGE→MRG→MGE→MGE→ARG→MGE

BS10_k97_66111	12	2 ARGs + 1 MRG + 9 MGEs co-localized within a 48,244 bp genomic region.	MGE→MGE→ARG→MGE→MGE→MGE→MGE→MGE→MGE→ARG→MGE→MRG
BS10_k97_7643	8	3 ARGs + 1 MRG + 4 MGEs co-localized within a 15,897 bp genomic region.	MRG→MGE→ARG→ARG→ARG→MGE→MGE→MGE
BS2_k97_11651	6	1 ARG + 1 MRG + 4 MGEs in tight linkage within a 13,268 bp genomic region.	MGE→MGE→MGE→MRG→ARG→MGE
BS2_k97_22671	4	1 ARG + 1 MRG + 2 MGEs in tight linkage within a 7,586 bp genomic region.	ARG→MRG→MGE→MGE
BS2_k97_28715	8	1 ARG + 2 MRGs + 5 MGEs in tight linkage within a 30,187 bp genomic region.	MGE→MGE→MGE→ARG→MRG→MGE→MRG→MGE
BS2_k97_30221	7	1 ARG + 1 MRG + 5 MGEs in tight linkage within a 9,977 bp genomic region.	MGE→ARG→MGE→MRG→MGE→MGE→MGE
BS2_k97_31401	3	1 ARG + 1 MRG + 1 MGE in tight linkage within a 4,269 bp genomic region.	MGE→ARG→MRG
BS2_k97_33535	10	1 ARG + 3 MRGs + 6 MGEs co-localized within a 22,472 bp genomic region.	MRG→MRG→MGE→MGE→MGE→ARG→MGE→MRG→MGE→MGE
BS2_k97_35332	8	2 ARGs + 1 MRG + 5 MGEs in tight linkage within a 26,048 bp genomic region.	MGE→MGE→MGE→MRG→MGE→ARG→ARG→MGE
BS2_k97_35873	3	1 ARG + 1 MRG + 1 MGE in tight linkage within a 9,160 bp genomic region.	MRG→ARG→MGE
BS2_k97_37049	6	2 ARGs + 1 MRG + 3 MGEs in tight linkage within a 16,438 bp genomic region.	MGE→MRG→MGE→ARG→ARG→MGE
BS2_k97_39161	5	1 ARG + 1 MRG + 3 MGEs in tight linkage within a 19,693 bp genomic region.	MGE→MGE→ARG→MRG→MGE
BS2_k97_44089	3	1 ARG + 1 MRG + 1 MGE in tight linkage within a 12,826 bp genomic region.	MRG→MGE→ARG

BS2_k97_44766	14	1 ARG + 1 MRG + 12 MGEs co-localized within a 42,494 bp genomic region.	MGE→ARG→MGE→MGE→MGE→MRG→MGE→MGE→MGE→MGE→MGE→MGE→MGE→MGE
BS2_k97_48414	5	1 ARG + 1 MRG + 3 MGEs in tight linkage within a 8,527 bp genomic region.	MGE→MGE→ARG→MGE→MRG
BS2_k97_53626	3	1 ARG + 1 MRG + 1 MGE in tight linkage within a 17,056 bp genomic region.	MRG→ARG→MGE
BS2_k97_56481	4	1 ARG + 1 MRG + 2 MGEs in tight linkage within a 6,030 bp genomic region.	MRG→ARG→MGE→MGE
BS2_k97_60734	4	1 ARG + 1 MRG + 2 MGEs in tight linkage within a 9,008 bp genomic region.	MGE→ARG→MGE→MRG
BS2_k97_61770	10	1 ARG + 2 MRGs + 7 MGEs co-localized within a 30,672 bp genomic region.	ARG→MGE→MRG→MGE→MRG→MGE→MGE→MGE→MGE→MGE
BS2_k97_6733	4	1 ARG + 1 MRG + 2 MGEs in tight linkage within a 14,720 bp genomic region.	MGE→MRG→MGE→ARG
BS2_k97_8056	4	2 ARGs + 1 MRG + 1 MGE in tight linkage within a 12,517 bp genomic region.	ARG→ARG→MRG→MGE
BS29_k97_10619	4	1 ARG + 1 MRG + 2 MGEs in tight linkage within a 12,469 bp genomic region.	MGE→MGE→MRG→ARG
BS29_k97_12994	4	1 ARG + 2 MRGs + 1 MGE in tight linkage within a 10,109 bp genomic region.	MRG→ARG→MRG→MGE
BS29_k97_13493	9	1 ARG + 1 MRG + 7 MGEs co-localized within a 56,003 bp genomic region.	ARG→MGE→MGE→MGE→MRG→MGE→MGE→MGE→MGE
BS29_k97_17331	3	1 ARG + 1 MRG + 1 MGE in tight linkage within a 3,255 bp genomic region.	MRG→ARG→MGE

BS29_k97_18240	5	1 ARG + 2 MRGs + 2 MGEs in tight linkage within a 19,005 bp genomic region.	MRG→MGE→ARG→MRG→MGE
BS29_k97_18672	9	2 ARGs + 2 MRGs + 5 MGEs in tight linkage within a 40,513 bp genomic region.	MGE→MRG→MRG→MGE→ARG→ARG→MGE →MGE→MGE
BS29_k97_19328	5	1 ARG + 1 MRG + 3 MGEs in tight linkage within a 10,996 bp genomic region.	MGE→ARG→MGE→MRG→MGE
BS29_k97_23808	3	1 ARG + 1 MRG + 1 MGE in tight linkage within a 8,414 bp genomic region.	ARG→MGE→MRG
BS29_k97_31274	8	1 ARG + 1 MRG + 6 MGEs in tight linkage within a 15,647 bp genomic region.	MGE→MGE→MGE→MRG→MGE→MGE→ARG →MGE
BS29_k97_38325	4	1 ARG + 1 MRG + 2 MGEs in tight linkage within a 7,023 bp genomic region.	MRG→MGE→MGE→ARG
BS29_k97_43598	53	1 ARG + 10 MRGs + 42 MGEs co-localized within a 250,641 bp genomic region.	MGE→MGE→MRG→MGE→MGE→MGE→MGE →MRG→MGE→MRG→MGE→MGE→MGE→M GE→MGE→MGE→MRG→MGE→MGE→MGE→ MGE→MGE→MGE→MGE→MGE→MGE→MGE →MGE→MGE→MRG→ARG→MGE→MGE→MR G→MGE→MRG→MGE→MGE→MRG→MGE→ MRG→MGE→MGE→MRG→MGE→MGE→MGE →MGE→MGE→MGE→MGE→MGE→MGE
BS29_k97_45521	5	1 ARG + 2 MRGs + 2 MGEs in tight linkage within a 14,377 bp genomic region.	MGE→MRG→MRG→MGE→ARG
BS29_k97_45765	3	1 ARG + 1 MRG + 1 MGE in tight linkage within a 3,691 bp genomic region.	MRG→MGE→ARG
BS29_k97_46991	24	1 ARG + 2 MRGs + 21 MGEs co-localized within a 125,185 bp genomic region.	MGE→MGE→MGE→MRG→MRG→MGE→MGE →MGE→MGE→MGE→MGE→ARG→MGE→MG

			E→MGE→MGE→MGE→MGE→MGE→MGE→MGE→MGE→MGE→MGE
BS29_k97_50527	19	1 ARG + 3 MRGs + 15 MGEs co-localized within a 108,135 bp genomic region.	MGE→MGE→MGE→MGE→MGE→MGE→MGE→MGE→ARG→MGE→MGE→MGE→MGE→MRG→MGE→MRG→MGE→MGE→MRG
BS29_k97_51056	3	1 ARG + 1 MRG + 1 MGE in tight linkage within a 11,285 bp genomic region.	MGE→ARG→MRG
BS29_k97_54116	17	1 ARG + 5 MRGs + 11 MGEs co-localized within a 96,951 bp genomic region.	MGE→MGE→MGE→MGE→MGE→MGE→MGE→MGE→MGE→MRG→MRG→ARG→MGE→MRG→MRG→MGE→MRG
BS29_k97_55478	3	1 ARG + 1 MRG + 1 MGE in tight linkage within a 6,347 bp genomic region.	MGE→ARG→MRG
BS29_k97_58411	18	4 ARGs + 3 MRGs + 11 MGEs co-localized within a 84,857 bp genomic region.	MGE→MRG→MRG→ARG→ARG→MGE→MGE→MGE→MGE→MGE→MGE→ARG→ARG→MGE→MGE→MGE→MGE→MRG
BS29_k97_61199	7	1 ARG + 1 MRG + 5 MGEs in tight linkage within a 12,368 bp genomic region.	MGE→ARG→MGE→MGE→MRG→MGE→MGE
BS29_k97_61751	8	1 ARG + 2 MRGs + 5 MGEs in tight linkage within a 28,236 bp genomic region.	MGE→MGE→MGE→MRG→MRG→MGE→MGE→ARG
BS29_k97_8092	5	1 ARG + 1 MRG + 3 MGEs in tight linkage within a 10,475 bp genomic region.	MGE→MRG→ARG→MGE→MGE
BS29_k97_9006	19	1 ARG + 2 MRGs + 16 MGEs co-localized within a 46,443 bp genomic region.	MGE→MGE→MGE→MGE→MGE→MGE→ARG→MGE→MRG→MGE→MGE→MGE→MGE→MRG→MGE→MGE→MGE→MGE→MGE
BS3_k97_16349	3	1 ARG + 1 MRG + 1 MGE in tight linkage within a 5,629 bp genomic region.	MGE→MRG→ARG

BS3_k97_21151	3	1 ARG + 1 MRG + 1 MGE in tight linkage within a 8,920 bp genomic region.	MRG→ARG→MGE
BS3_k97_23670	4	2 ARGs + 1 MRG + 1 MGE in tight linkage within a 10,211 bp genomic region.	MRG→ARG→ARG→MGE
BS3_k97_2883	3	1 ARG + 1 MRG + 1 MGE in tight linkage within a 3,377 bp genomic region.	MRG→MGE→ARG
BS3_k97_29701	8	1 ARG + 1 MRG + 6 MGEs co-localized within a 13,644 bp genomic region.	MRG→ARG→MGE→MGE→MGE→MGE→MGE →MGE
BS32_k97_17145	6	1 ARG + 1 MRG + 4 MGEs in tight linkage within a 11,789 bp genomic region.	MGE→ARG→MGE→MRG→MGE→MGE
BS32_k97_26532	5	1 ARG + 1 MRG + 3 MGEs in tight linkage within a 6,588 bp genomic region.	MGE→MGE→ARG→MGE→MRG
BS32_k97_33604	7	1 ARG + 1 MRG + 5 MGEs in tight linkage within a 19,186 bp genomic region.	MGE→ARG→MRG→MGE→MGE→MGE→MGE
BS32_k97_35611	3	1 ARG + 1 MRG + 1 MGE in tight linkage within a 7,949 bp genomic region.	MGE→MRG→ARG
BS32_k97_36541	5	1 ARG + 2 MRGs + 2 MGEs in tight linkage within a 15,731 bp genomic region.	MRG→MGE→MRG→MGE→ARG
BS32_k97_39879	3	1 ARG + 1 MRG + 1 MGE in tight linkage within a 4,447 bp genomic region.	ARG→MGE→MRG
BS32_k97_44320	3	1 ARG + 1 MRG + 1 MGE in tight linkage within a 13,793 bp genomic region.	MRG→MGE→ARG
BS32_k97_44323	5	1 ARG + 1 MRG + 3 MGEs in tight linkage within a 21,545 bp genomic region.	MRG→ARG→MGE→MGE→MGE
BS32_k97_52192	4	1 ARG + 1 MRG + 2 MGEs in tight linkage within a 20,355 bp genomic region.	MGE→MGE→MRG→ARG

BS32_k97_53752	3	1 ARG + 1 MRG + 1 MGE in tight linkage within a 4,121 bp genomic region.	MGE→MRG→ARG
BS32_k97_53785	9	1 ARG + 2 MRGs + 6 MGEs in tight linkage within a 41,938 bp genomic region.	MGE→MGE→MGE→MGE→MGE→ARG→MRG →MRG→MGE
BS32_k97_54862	4	2 ARGs + 1 MRG + 1 MGE in tight linkage within a 2,646 bp genomic region.	MRG→ARG→MGE→ARG
BS32_k97_77355	10	4 ARGs + 1 MRG + 5 MGEs in tight linkage within a 36,950 bp genomic region.	MGE→MGE→ARG→ARG→ARG→ARG→MGE →MGE→MRG→MGE
BS32_k97_82179	16	3 ARGs + 4 MRGs + 9 MGEs co-localized within a 73,204 bp genomic region.	MGE→MGE→MGE→MRG→MRG→MGE→MGE →MGE→MGE→MGE→MRG→ARG→ARG→AR G→MGE→MRG
BS32_k97_82574	13	1 ARG + 1 MRG + 11 MGEs co-localized within a 53,978 bp genomic region.	MGE→ARG→MGE→MGE→MGE→MGE→MGE →MGE→MGE→MGE→MGE→MGE→MRG
BS9_k97_11091	13	2 ARGs + 2 MRGs + 9 MGEs co-localized within a 20,142 bp genomic region.	MGE→MGE→MGE→MGE→MGE→MGE→MGE →MGE→ARG→MRG→MRG→ARG→MGE
BS9_k97_11886	5	1 ARG + 1 MRG + 3 MGEs in tight linkage within a 14,990 bp genomic region.	ARG→MGE→MGE→MRG→MGE
BS9_k97_12515	5	2 ARGs + 1 MRG + 2 MGEs in tight linkage within a 14,727 bp genomic region.	MGE→ARG→MGE→MRG→ARG
BS9_k97_13320	4	2 ARGs + 1 MRG + 1 MGE in tight linkage within a 7,106 bp genomic region.	MGE→MRG→ARG→ARG
BS9_k97_13764	4	2 ARGs + 1 MRG + 1 MGE in tight linkage within a 9,908 bp genomic region.	MGE→MRG→ARG→ARG
BS9_k97_15185	5	2 ARGs + 1 MRG + 2 MGEs in tight linkage within a 6,828 bp genomic region.	MRG→ARG→MGE→MGE→ARG

BS9_k97_27488	6	2 ARGs + 2 MRGs + 2 MGEs in tight linkage within a 7,234 bp genomic region.	MRG→ARG→MGE→ARG→MRG→MGE
BS9_k97_29617	8	2 ARGs + 1 MRG + 5 MGEs in tight linkage within a 8,812 bp genomic region.	ARG→MGE→ARG→MGE→MGE→MRG→MGE →MGE
BS9_k97_31380	5	1 ARG + 1 MRG + 3 MGEs in tight linkage within a 16,789 bp genomic region.	MGE→MGE→MRG→ARG→MGE
BS9_k97_6889	5	1 ARG + 1 MRG + 3 MGEs in tight linkage within a 11,883 bp genomic region.	ARG→MGE→MGE→MGE→MRG
BS9_k97_7663	6	2 ARGs + 1 MRG + 3 MGEs in tight linkage within a 9,359 bp genomic region.	ARG→ARG→MGE→MGE→MRG→MGE
YH13_k97_56926	3	1 ARG + 1 MRG + 1 MGE in tight linkage within a 1,995 bp genomic region.	ARG→MGE→MRG
YY11_k97_111168	3	1 ARG + 1 MRG + 1 MGE in tight linkage.	MRG → MGE → ARG
YY11_k97_112085	3	1 ARG + 1 MRG + 1 MGE in tight linkage.	MRG → ARG → MGE
YY11_k97_112207	5	1 ARG + 2 MRGs + 2 MGEs in tight linkage within a 9,751 bp genomic region.	MRG→MRG→ARG→MGE→MGE
YY11_k97_112284	18	2 ARGs + 4 MRGs + 12 MGEs co-localized within a 56026 bp region.	MGE → MGE → MRG → MGE → MGE → MGE → MGE → MGE → MGE → MGE → MRG → MRG → MGE → MRG → MGE → ARG → ARG → MGE
YY11_k97_112731	9	1 ARG + 2 MRGs + 6 MGEs co-localized within a 27558 bp region.	MGE → MGE → MGE → MGE → MGE → MRG → MRG → ARG → MGE
YY11_k97_127885	10	1 ARG + 2 MRGs + 7 MGEs co-localized within a 33422 bp region.	MGE → MGE → MGE → MRG → MGE → MGE → MRG → MGE → ARG → MGE
YY11_k97_12988	7	1 ARG + 1 MRG + 5 MGEs in tight linkage.	MGE → MGE → MRG → MGE → MGE → ARG → MGE

YY11_k97_155442	5	2 ARGs + 1 MRG + 2 MGEs in tight linkage.	MGE → MRG → ARG → ARG → MGE
YY11_k97_15889	6	2 ARGs + 1 MRG + 3 MGEs in tight linkage.	MGE → ARG → ARG → MRG → MGE → MGE
YY11_k97_161834	3	1 ARG + 1 MRG + 1 MGE in tight linkage.	MRG → ARG → MGE
YY11_k97_37280	3	1 ARG + 1 MRG + 1 MGE in tight linkage.	ARG → MGE → MRG
YY11_k97_43613	4	2 ARGs + 1 MRG + 1 MGE in tight linkage.	ARG → MRG → ARG → MGE
YY11_k97_44315	3	1 ARG + 1 MRG + 1 MGE in tight linkage.	MGE → ARG → MRG
YY11_k97_57487	4	1 ARG + 1 MRG + 2 MGEs in tight linkage.	ARG → MGE → MGE → MRG
YY11_k97_58405	7	1 ARG + 3 MRGs + 3 MGEs in tight linkage.	MRG → MGE → ARG → MGE → MGE → MRG → MRG
YY11_k97_65980	17	4 ARGs + 1 MRG + 12 MGEs co-localized within a 75,341 bp genomic region.	MGE→MRG→ARG→MGE→MGE→MGE→MGE →MGE→MGE→MGE→MGE→ARG→MGE→AR G→ARG→MGE→MGE
YY11_k97_66638	8	2 ARGs + 1 MRG + 5 MGEs co-localized within a 33989 bp region.	ARG → MGE → MGE → MRG → MGE → MGE → MGE → ARG
YY11_k97_67232	5	2 ARGs + 1 MRG + 2 MGEs in tight linkage within a 6,385 bp genomic region.	MGE→MGE→ARG→MRG→ARG
YY11_k97_70765	4	1 ARG + 1 MRG + 2 MGEs in tight linkage within a 13,335 bp genomic region.	MRG→ARG→MGE→MGE
YY11_k97_7543	11	1 ARG + 2 MRGs + 8 MGEs co-localized within a 19334 bp region.	MGE → MRG → MRG → MGE → MGE → MGE → MGE → MGE → MGE → ARG → MGE
YY11_k97_83978	8	1 ARG + 2 MRGs + 5 MGEs co-localized within a 34713 bp region.	MGE → MRG → MGE → MRG → ARG → MGE → MGE → MGE
YY11_k97_88036	8	1 ARG + 1 MRG + 6 MGEs in tight linkage.	MGE → ARG → MRG → MGE → MGE → MGE → MGE → MGE
YY11_k97_96345	5	1 ARG + 1 MRG + 3 MGEs in tight linkage.	MGE → MRG → MGE → ARG → MGE

YY18_k97_11716	25	3 ARGs + 4 MRGs + 18 MGEs co-localized within a 112803 bp region.	MGE → MRG → MRG → MGE → ARG → MGE → MGE → MRG → MGE → MGE → ARG → MGE → MGE → MGE → MGE → MGE → MGE → MGE → MRG → MGE → ARG → MGE → MGE → MGE → MGE
YY18_k97_12388	9	1 ARG + 1 MRG + 7 MGEs co-localized within a 34356 bp region.	MGE → MGE → ARG → MGE → MRG → MGE → MGE → MGE → MGE
YY18_k97_12572	5	1 ARG + 1 MRG + 3 MGEs co-localized within a 19231 bp region.	MGE → ARG → MGE → MGE → MRG
YY18_k97_13932	5	1 ARG + 1 MRG + 3 MGEs co-localized within a 10473 bp region.	MGE → MGE → ARG → MRG → MGE
YY18_k97_14262	17	1 ARG + 1 MRG + 15 MGEs co-localized within a 67594 bp region.	MGE → MGE → MGE → MGE → MGE → MGE → MGE → MGE → MGE → MGE → MGE → MGE → MGE → ARG → MGE → MGE → MRG
YY18_k97_1599	12	4 ARGs + 1 MRG + 7 MGEs co-localized within a 37231 bp region.	ARG → ARG → ARG → MGE → MGE → MGE → MGE → MRG → ARG → MGE → MGE → MGE
YY18_k97_18096	13	2 ARGs + 1 MRG + 10 MGEs co-localized within a 44680 bp region.	MGE → MGE → MGE → ARG → ARG → MGE → MGE → MGE → MGE → MGE → MGE → MRG → MGE
YY18_k97_18818	5	1 ARG + 1 MRG + 3 MGEs co-localized within a 9840 bp region.	MGE → ARG → MRG → MGE → MGE
YY18_k97_18828	14	2 ARGs + 1 MRG + 11 MGEs co-localized within a 54115 bp region.	ARG → MGE → MGE → ARG → MGE → MGE → MGE → MGE → MGE → MGE → MGE → MGE → MRG → MGE
YY18_k97_18865	9	1 ARG + 1 MRG + 7 MGEs co-localized within a 78079 bp region.	MGE → MGE → MGE → MGE → MGE → ARG → MGE → MGE → MRG

YY18_k97_19002	22	2 ARGs + 2 MRGs + 18 MGEs co-localized within a 102230 bp region.	MGE → ARG → MGE → MRG → MGE → MGE → MGE → MGE → MGE → MGE → MGE → MGE → MGE → MRG → MGE → MGE → MGE → MGE → ARG → MGE → MGE → MGE
YY18_k97_4754	11	1 ARG + 1 MRG + 9 MGEs co-localized within a 48001 bp region.	MGE → MGE → MGE → MGE → MGE → MRG → MGE → MGE → ARG → MGE → MGE
YY18_k97_9391	17	1 ARG + 4 MRGs + 12 MGEs co-localized within a 74728 bp region.	MGE → MGE → MGE → MRG → MGE → MGE → MGE → MGE → MGE → MGE → MRG → MRG → ARG → MGE → MGE → MRG → MGE
YY21_k97_17402	11	1 ARG + 2 MRGs + 8 MGEs co-localized within a 19051 bp region.	MGE → ARG → MGE → MGE → MGE → MGE → MGE → MGE → MRG → MRG → MGE
YY5_k97_140682	10	1 ARG + 1 MRG + 8 MGEs co-localized within a 46,707 bp genomic region.	MGE→MRG→MGE→MGE→MGE→MGE→MGE→ARG→MGE→MGE
YY5_k97_161534	23	1 ARG + 1 MRG + 21 MGEs co-localized within a 97,752 bp genomic region.	MGE→MGE→MGE→MGE→ARG→MGE→MGE→MGE→MGE→MGE→MGE→MGE→MGE→MGE→MGE→MGE→MGE→MGE→MGE→MGE→MRG→MGE→MGE
YY5_k97_178925	7	1 ARG + 1 MRG + 5 MGEs in tight linkage within a 32,268 bp genomic region.	MGE→MRG→MGE→MGE→ARG→MGE→MGE
YY5_k97_182783	15	1 ARG + 2 MRGs + 12 MGEs co-localized within a 43,894 bp genomic region.	MGE→MGE→MGE→MGE→MGE→MRG→MGE→MGE→MGE→MGE→MRG→MGE→MGE→MGE→ARG
YY5_k97_197055	27	4 ARGs + 4 MRGs + 19 MGEs co-localized within a 125,786 bp genomic region.	MGE→ARG→MRG→MRG→MGE→ARG→MGE→MGE→MGE→MGE→MGE→MGE→MGE→MGE→MRG→MGE→MGE→MGE→MGE→MGE→MGE→MRG→MGE→ARG→MGE→MGE→ARG

YY5_k97_204647	28	3 ARGs + 3 MRGs + 22 MGEs co-localized within a 189,657 bp genomic 区域。	MGE→ARG→MRG→MGE→MGE→MRG→MGE→MGE→MRG→MGE→MGE→MGE→ARG→ARG→MGE→MGE→MGE→MGE→MGE→MGE→MGE→MGE→MGE→MGE→MGE→MGE→MGE→MGE
YY5_k97_211846	8	2 ARGs + 1 MRG + 5 MGEs in tight linkage within a 23,220 bp genomic region.	MGE→MGE→MGE→ARG→MRG→MGE→MGE→ARG
YY5_k97_28958	6	1 ARG + 2 MRGs + 3 MGEs in tight linkage within a 26,016 bp genomic region.	MGE→MGE→ARG→MGE→MRG→MRG
YY5_k97_48311	24	4 ARGs + 4 MRGs + 16 MGEs co-localized within a 145,362 bp genomic region.	MRG→MRG→ARG→MGE→MGE→MGE→MGE→ARG→ARG→MGE→MRG→MGE→ARG→MRG→MGE→MGE→MGE→MGE→MGE→MGE→MGE→MGE→MGE→MGE
YY5_k97_58277	3	1 ARG + 1 MRG + 1 MGE in tight linkage within a 2,557 bp genomic region.	ARG→MGE→MRG
YY5_k97_59156	12	1 ARG + 1 MRG + 10 MGEs co-localized within a 66,869 bp genomic region.	MGE→MGE→MGE→ARG→MGE→MGE→MGE→MRG→MGE→MGE→MGE→MGE
YY5_k97_81589	11	3 ARGs + 1 MRG + 7 MGEs co-localized within a 88,047 bp genomic 区域。	ARG→ARG→MGE→MGE→MGE→ARG→MGE→MGE→MGE→MRG→MGE
YY8_k97_14573	16	3 ARGs + 1 MRG + 12 MGEs co-localized within a 33900 bp region.	MGE → MGE → ARG → (MGE cluster) → MRG → ARG → (MGE cluster) → ARG
YY8_k97_15572	11	3 ARGs + 3 MRGs + 5 MGEs co-localized within a 21900 bp region.	ARG → MRG → MGE → MGE → MRG → MRG → MGE → MGE → ARG → MGE → ARG
YY8_k97_15949	4	1 ARG + 1 MRG + 2 MGEs co-localized within a 7200 bp region.	ARG → MGE → MGE → MRG

YY8_k97_15954	3	1 ARG + 1 MRG + 1 MGE in tight linkage within a 17900 bp region.	ARG → MGE → MRG
YY8_k97_16314	9	2 ARGs + 2 MRGs + 5 MGEs co-localized within a 22900 bp region.	MRG → MGE → ARG → (MGE cluster) → MRG → MGE → ARG
YY8_k97_17523	3	1 ARG + 1 MRG + 1 MGE in tight linkage within a 9500 bp region.	MGE → ARG → MRG
YY8_k97_18126	16	1 ARG + 2 MRGs + 13 MGEs co-localized within a 5710 bp region.	MGE → MGE → MRG → (MGE cluster) → ARG → (MGE cluster) → MRG
YY8_k97_1927	4	1 ARG + 1 MRG + 2 MGEs co-localized within a 17700 bp region.	MGE → MRG → ARG → MGE
YY8_k97_23897	13	1 ARG + 3 MRGs + 9 MGEs co-localized within a 73100 bp region.	MRG → (MGE cluster) → ARG → (MGE cluster) → MRG → (MGE cluster) → MRG
YY8_k97_24141	11	5 ARGs + 1 MRG + 5 MGEs co-localized within a 37300 bp region.	MGE → ARG → ARG → ARG → MGE → MGE → MRG → MGE → MGE → ARG → ARG
YY8_k97_28251	3	1 ARG + 1 MRG + 1 MGE in tight linkage within a 9200 bp region.	MGE → ARG → MRG
YY8_k97_30987	9	2 ARGs + 2 MRGs + 5 MGEs co-localized within a 19500 bp region.	MGE → ARG → ARG → (MGE cluster) → MRG → MRG → MGE
YY8_k97_37937	14	1 ARG + 1 MRG + 12 MGEs co-localized within a 35400 bp region.	(MGE cluster) → MRG → (MGE cluster) → ARG → (MGE cluster)
YY8_k97_39859	7	1 ARG + 2 MRGs + 4 MGEs co-localized within a 36100 bp region.	MGE → MRG → ARG → MGE → MRG → MGE → MGE
YY8_k97_41456	7	3 ARGs + 1 MRG + 3 MGEs co-localized within a 28100 bp region.	MGE → MRG → ARG → ARG → ARG → MGE → MGE
YY8_k97_42515	6	1 ARG + 1 MRG + 4 MGEs co-localized within a 6600 bp region.	MGE → ARG → MGE → MGE → MRG → MGE

YY8_k97_42682	11	1 ARG + 3 MRGs + 7 MGEs co-localized within a 21500 bp region.	(MGE cluster) → ARG → MRG → MRG → MGE → MRG → (MGE cluster)
YY8_k97_42966	8	1 ARG + 1 MRG + 6 MGEs co-localized within a 28200 bp region.	MGE → MRG → MGE → ARG → (MGE cluster)
YY8_k97_9139	5	1 ARG + 2 MRGs + 2 MGEs co-localized within a 14900 bp region.	MGE → MGE → MRG → MRG → ARG
YY8_k97_9945	13	1 ARG + 4 MRGs + 8 MGEs co-localized within a 45300 bp region.	MGE → MGE → MRG → (MGE cluster) → MRG → MRG → ARG → MRG → (MGE cluster)
